# The Impact of Local Genome Sequence on Defining Heterochromatin Domains

**DOI:** 10.1371/journal.pgen.1000453

**Published:** 2009-04-10

**Authors:** Bayly S. Wheeler, Jared A. Blau, Huntington F. Willard, Kristin C. Scott

**Affiliations:** Duke Institute for Genome Sciences and Policy, Duke University, Durham, North Carolina, United States of America; Medical Research Council Human Genetics Unit, United Kingdom

## Abstract

Characterizing how genomic sequence interacts with *trans*-acting regulatory factors to implement a program of gene expression in eukaryotic organisms is critical to understanding genome function. One means by which patterns of gene expression are achieved is through the differential packaging of DNA into distinct types of chromatin. While chromatin state exerts a major influence on gene expression, the extent to which *cis*-acting DNA sequences contribute to the specification of chromatin state remains incompletely understood. To address this, we have used a fission yeast sequence element (L5), known to be sufficient to nucleate heterochromatin, to establish *de novo* heterochromatin domains in the *Schizosaccharomyces pombe* genome. The resulting heterochromatin domains were queried for the presence of H3K9 di-methylation and Swi6p, both hallmarks of heterochromatin, and for levels of gene expression. We describe a major effect of genomic sequences in determining the size and extent of such *de novo* heterochromatin domains. Heterochromatin spreading is antagonized by the presence of genes, in a manner that can occur independent of strength of transcription. Increasing the dosage of Swi6p results in increased heterochromatin proximal to the L5 element, but does not result in an expansion of the heterochromatin domain, suggesting that in this context genomic effects are dominant over *trans* effects. Finally, we show that the ratio of Swi6p to H3K9 di-methylation is sequence-dependent and correlates with the extent of gene repression. Taken together, these data demonstrate that the sequence content of a genomic region plays a significant role in shaping its response to encroaching heterochromatin and suggest a role of DNA sequence in specifying chromatin state.

## Author Summary

Epigenetic packaging of DNA sequence into chromatin is a major force in shaping the function of complex genomes. Different types of chromatin have distinct effects on gene expression, and thus chromatin state imparts distinct features on the associated genomic DNA. Our study focuses on the transition between two opposing chromatin states: euchromatin, which generally correlates with gene expression, and heterochromatin, which is typically refractive to gene expression. While heterochromatin is capable of spreading into euchromatic domains, the parameters that influence such spreading are unknown. We established heterochromatin at ectopic sites in the genome and evaluated whether specific DNA sequences affected the extent of heterochromatin spreading and the transition between heterochromatin and euchromatin. We found that the nature of the genomic DNA neighboring the heterochromatic sequence dramatically affected the extent of heterochromatin spreading. In particular, the presence of genes antagonized the spread of heterochromatin, whereas neutral sequence elements were incorporated into the domain. This study demonstrates that genome sequence and chromatin identity are inextricably linked; features of both interact to determine the structural and functional fate of underlying DNA sequences.

## Introduction

Correct patterns of gene expression are established by orchestrated interactions among *cis*-regulatory elements, *trans*-acting factors and the surrounding chromatin environment. How these interactions are coordinated and to what extent genomic sequence serves as a blueprint, directing these interactions towards normal growth and development, remain major questions in genome biology.

Chromatin has classically been divided into two functionally distinct types: heterochromatin and euchromatin. Genes inserted within, or proximal to, major heterochromatin domains can exhibit either variegated or complete silencing [1]–[7]. This repression, referred to as position effect variegation (PEV), results from the propagation of heterochromatin marks along the chromosome, placing the euchromatic gene into a chromatin context that is incompatible with normal gene expression [2],[8],[9]. While PEV and the factors that contribute to it have been most thoroughly elucidated in yeast and flies, position-dependent gene silencing has been observed in a range of organisms including both mice and humans [1]–[3],[10]. Indeed, there are examples of human disease that can be attributed to gene silencing associated with aberrant formation of heterochromatin [11]–[14]. Together, these studies highlight the important relationship between chromatin context and gene expression and suggest that eukaryotes have developed mechanisms to counter the spread of repressive heterochromatin [2],[8],[15],[16]. However, the nature of these mechanisms and the extent to which they utilize specific DNA sequences remains incompletely understood.

Several studies have pointed towards the importance of genome sequence in shaping epigenetic states. For example, insulators are specific DNA sequences that protect genes from the regulatory effects of neighboring domains, thus enforcing domain boundaries [17]. As presently defined, insulator activity has two components: the ability to prevent cross-talk between an enhancer and promoter (enhancer blockers) and the ability to stop the spread of repressive heterochromatin (heterochromatin barriers) [17]–[20]. First identified and characterized in flies [18],[21], insulators have since been identified in vertebrates [22]–[24].

Elucidating the role of genome sequence in shaping chromatin domains requires an experimental system in which heterochromatin nucleation can be initiated in a controlled manner. To this end, we have examined heterochromatin spreading from a *de novo* nucleation site in the fission yeast, *Schizosaccharomyces pombe*. The unique advantage of this system, in addition to its genetic tractability, is the presence of well-defined DNA sequences, referred to here as heterochromatin-nucleating sequences, that are sufficient to induce heterochromatin formation *de novo* [25]–[27]. Moreover, introduction of a *de novo* heterochromatin domain at a euchromatic locus permits a simplified view of this process, in contrast to native domains of heterochromatin that result from the complex interplay of multiple sites of nucleation and heterochromatin barriers [27]–[29]. Analysis of the resulting *de novo* heterochromatin domains clearly implicates primary DNA sequence in defining both the magnitude and extent of the heterochromatin domain. The conceptual framework that emerges from this study provides a basis for exploring the nature of complex genomes and the impact of genome sequence on the establishment and maintenance of chromatin domains, in organisms ranging from yeast to mammals.

## Results

### The L5 element nucleates a *de novo* heterochromatin domain encompassing adjacent genomic sequences

Previous studies in *S. pombe* have demonstrated that a fragment of pericentromeric DNA, called L5, is capable of nucleating heterochromatin, marked by di-methylation at H3K9 (H3K9me2) and the presence of the HP1 homologue, Swi6p, at an ectopic site through an RNAi-dependent mechanism [25],[30]. Integration of the L5 element leads to the repression of an adjacent reporter gene in a manner that appears largely similar to that observed at the endogenous centromere [25],[27],[30]. What is unknown, however, is the extent to which L5-nucleated heterochromatin is capable of extending past the reporter construct into endogenous genomic sequences.

To address this question, we created a construct containing the 1.6 kb L5 element upstream of an *ade6^+^* reporter gene. This construct was then integrated at the euchromatic *ura4* ^+^ locus in order to create *ura4*::L5-*ade6* ^+^ strains. In addition to the L5-containing construct, a control construct bearing only the *ade6^+^* gene was also integrated at the *ura4^+^* locus (*ura4*::*ade6^+^*). The effect of L5-integration on the chromatin environment of sequences within the *ura4* locus was characterized by quantifying H3K9me2 and Swi6p levels throughout the region using chromatin immunoprecipitation (ChIP).

**Figure 1:**
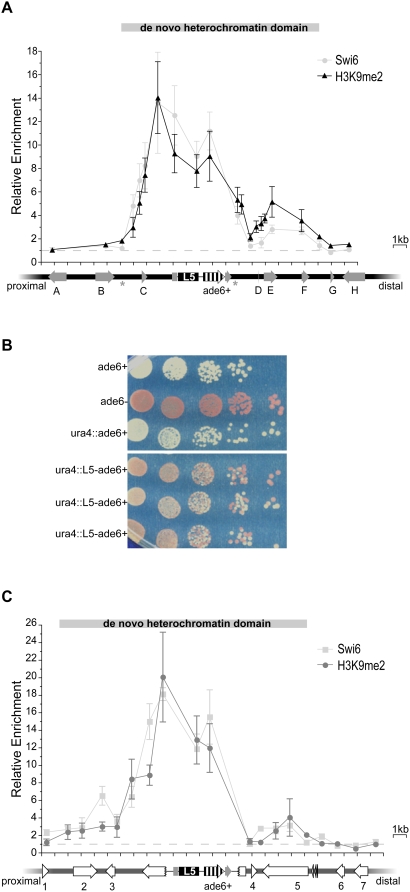
L5 initiates formation of *de novo* heterochromatin domains at two distinct loci. (A) Levels of H3K9me2 (black) and Swi6p (grey) were assayed via ChIP and are shown relative to levels at *act1^+^*, such that the dotted line indicates no enrichment. The enrichment data are plotted versus the *ura4* genomic region; grey arrows represent genes, each given a letter identifier, and the direction of the arrowhead indicates the direction of transcription. Non-coding RNAs are shown as asterisks. The disrupted *ura4* gene is shown as a broken arrow surrounding L5 and *ade6^+^*. Tick marks are spaced every 1 kb. The *de novo* heterochromatin domain is defined as regions that are greater than 2-fold enriched in H3K9me2. Error bars represent the standard error of the mean. (B) *ade6^+^* expression is reduced in strains containing L5. Shown is the phenotypic assessment of *ade6*+ expression using a serial dilution assay. Each row represents an individual strain plated on adenine limiting media. *ade6^+^* and *ade6^−^* strains demonstrate the phenotypic effects of *ade6* expression and are compared to a representative *ura4::ade6* ^+^ control strain and three independent *ura4*::L5-*ade6^+^* strains. (C) H3K9me2 and Swi6p enrichment are plotted in dark and light grey, respectively, at the *spbc2f12.03* locus. Genes in this genomic location are labeled numerically and are represented by open arrows. The disrupted *spcbc2f12.03* gene is represented by a broken arrow.

In the presence of the L5 element, H3K9me2 was enriched ∼2- to >10-fold over both the reporter gene and the surrounding genomic neighborhood (Figure 1A and Figure S1A), extending 4 kb proximal and 10 kb distal to L5. The pattern of Swi6p enrichment is remarkably similar to the level of H3K9me2, consistent with previous reports demonstrating that H3K9me2 and Swi6p have tightly overlapping distributions within heterochromatin domains (Figure 1A and Figure S1A) [28]. These data demonstrate that heterochromatin assembly is not limited to the L5-element and the reporter gene; instead, heterochromatin spreads bi-directionally into adjacent, formerly euchromatic, sequences, resulting in a *de novo* heterochromatin domain that spans approximately 15 kb. Throughout, we will describe the properties of a heterochromatin domain by its extent, the distance over which heterochromatin is enriched, and its magnitude, the level of heterochromatin enrichment at a given location.

### The presence of heterochromatin causes reduced expression within the *de novo* domain

Because the chromatin state of genes near *ura4* changes upon insertion of L5, we sought to determine whether gene expression at the ectopic locus was also altered. Quantitative RT- PCR (qRT–PCR) was used to quantify the levels of mRNA in the presence of L5 relative to control strains lacking L5. As expected from earlier studies [25], we observed an L5-dependent decrease in *ade6^+^* expression; however, the reduction in expression was moderate (34±5%), indicating that silencing is incomplete in these strains (Table 1). In addition to *ade6^+^*, two genes within the *de novo* heterochromatin domain, located 2.7 kb proximal and 4.9 kb distal from the L5 element, also exhibited a decrease in expression in the presence of L5, 43±10% and 52±8%, respectively. Gene expression outside of the *de novo* heterochromatin domain was also analyzed (Figure 1A). As predicted, three genes (A, B, H) had no significant difference in transcript abundance in the presence of L5 (Table 1). The remaining gene, G, as well as gene F that lies within the *de novo* domain, exhibit a discordant relationship between the enrichment of heterochromatin marks and the level of gene expression. Together, these results suggest that gene-specific features may have a greater influence on the level of gene repression, as compared to the centromere, where gene repression is more complete [31].

**Table 1:** *de novo* heterochromatin domains are associated with reduced gene expression at both the *ura4* and *spbc2f12.03* loci.

Gene^1^	Gene name	Distance from L5 (bp)^2^	Relative mRNA (+L5/−L5)	N^3^	p^4^
A	alg11^5^	8393	1.02±0.11	8	0.853
B	CC330.07	6245	1.21±0.10	11	0.101
C	spcc330.06	2723	0.57±0.10	14	**0.002**
	ade6	340	0.67±0.05	13	**0.000**
D	tDNAgly	4535			
E	mug135	4943	0.48±0.08	8	**0.001**
F	CC330.03	7819	0.90±0.20	17	0.628
G	CC330.19	9984	0.66±0.03	8	**0.008**
H	rhp7	10902	1.08±0.15	4	0.731
1	mlo3^6^	10609	0.83±0.09	5	0.172
2	byr2	8193	0.67±0.15	6	0.284
3	mrpl7	4875	0.81±0.15	6	0.289
	ade6	340	0.48±0.10	5	**0.001**
4	rpl1701	4028	0.58±0.08	5	**0.007**
5	BC2f12.05	9177	0.62±0.18	6	0.087
6	rpl802	11334	1.03±0.08	5	0.752
7	ceg1^7^	13164	1.08±0.11	7	0.335

1 From Figure 1A and 1C.

2 Distance of the translation start site from the nearest edge of L5.

3 Number of independent RNA isolations included in analysis.

4 p-value resulting from comparison between *ura4*::*ade6+* and *ura4*::L5-*ade6+* strains.

5 Essential for viability [82].

6 Mutation in mlo3 results in a growth defect [83].

7 Essential for viability [84].

Relative gene expression in the *ura4* and *spbc2f12.03* loci. Steady-state mRNA for genes in the *ura4* (A–H) and *spbc2f12.03* (1–7) loci were assayed via quantitative RT–PCR and are reported relative to *ura4*::*ade6^+^* control strains. The distance from the transcription start site to the nearest edge of L5 is indicated for each gene.

To further explore the extent of *ade6^+^*silencing, we utilized a phenotypic assay for *ade6^+^*expression. This assay allows the extent of silencing to be resolved on a sub-colony level, as opposed to the population level queried by qRT–PCR. Under conditions of limiting adenine, yeast that are mutant, or silenced [1], for *ade6^+^* accumulate a metabolic intermediate that results in red pigmentation. In contrast, cells in which *ade6^+^*is expressed at wild type levels remain white. Results from the phenotypic assay indicate that there is significant heterogeneity among colonies in the *ura4*::L5-*ade6* ^+^ strains (Figure 1B). Similar to classic PEV, the colony phenotypes ranged from white to red [8]. However, distinct from PEV in *Drosophila*, we also observed intermediate phenotypes of pink and red with white sectors, consistent with PEV as observed in yeast [1],[32],[33].

### *de novo* heterochromatin domains are sensitive to genomic location

We next wanted to determine whether the magnitude and extent of a *de novo* heterochromatin domain depends upon its location in the *S. pombe* genome or whether domain properties are intrinsic to the L5 element itself. To explore this, we identified a second integrant of the *ura4*::L5-*ade6^+^*construct on chromosome 2 (*spbc2f12.03*::*ura4*::L5-*ade6^+^*). Comparison of H3K9me2 and Swi6p enrichment at L5 and *ade6^+^* between the two sites of integration reveals similar patterns of enrichment, suggesting that the nucleation of heterochromatin and local spreading are not sensitive to the changes in genomic location from *ura4* to *spbc2f12.03* (Figure 1C).

We next compared the magnitude and extent of the *de novo* heterochromatin domains formed at these two genomic locations. Distal to L5, the patterns of heterochromatin enrichment are markedly similar between the *ura4* and *spbc2f12.03* loci. In contrast, heterochromatin is observed 9 kb proximal to L5 at the *spbc2f12.03* locus as compared to only 4.9 kb at the *ura4* locus (compare Figure 1A and 1C). While this expansion at the *spbc2f12.03* locus is only modestly enriched in H3K9me2, it is also marked by the presence of Swi6p (Figure 1C), suggesting there is a 4.1 kb expansion of the heterochromatin domain relative to the *ura4* locus. Thus, the proximal boundary of the *de novo* heterochromatin domain at the *ura4* locus is influenced by genomic location as opposed to reflecting an intrinsic limitation of the L5-element.

To determine whether the *de novo* heterochromatin domain at *spbc2f12.03* alters gene expression, qRT–PCR was used to analyze mRNA levels at the *spbc2f12.03* ectopic locus. Repression is observed at *ade6^+^* and the nearby *rpl1701^+^* gene, but not genes 2, 3 and 5 (Figure 1C, gene 4; Table 1). Thus, analogous to gene F at the *ura4* locus, the recruitment of heterochromatic marks to an ectopic locus is not always associated with significant gene silencing.

### *Cis*-acting sequences shape the *de novo* heterochromatin domain

The experiments described above demonstrate that the extent of an L5-dependent *de novo* heterochromatin domain can vary between different locations in the genome. To explore whether these differences are attributable to *cis*-acting factors, we engineered constructs in which different DNA sequences were placed adjacent to L5. These constructs were then inserted at the *ura4* locus to examine the role of sequence in defining the heterochromatin domain, without altering its location in the genome.

**Figure 2:**
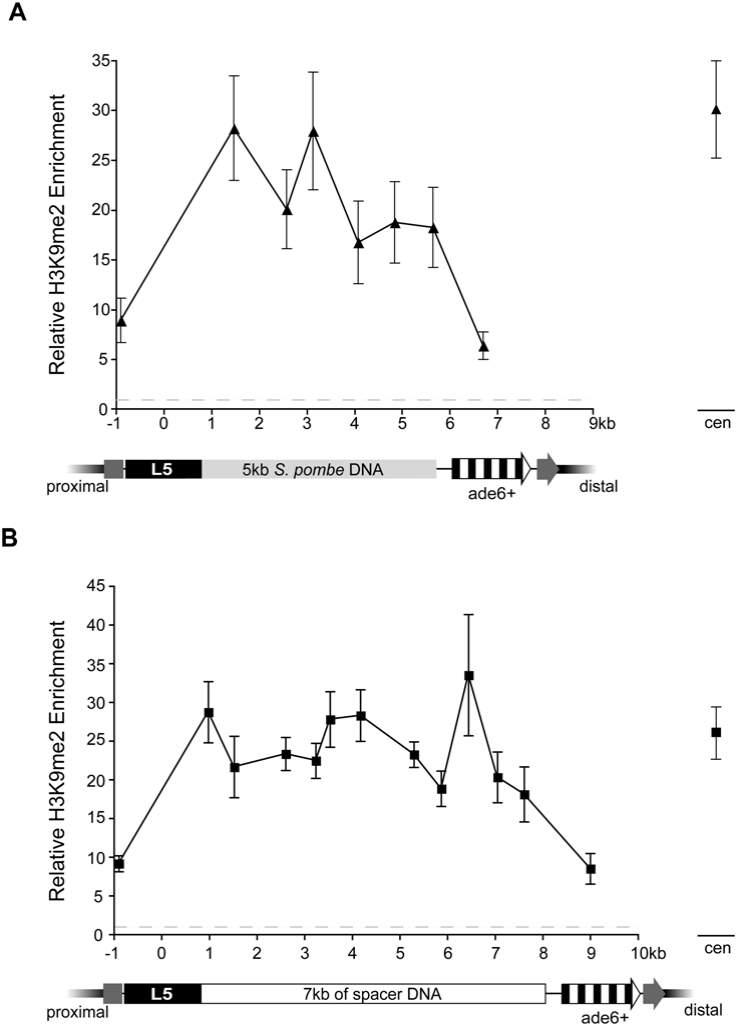
H3K9me2 is highly enriched over intergenic and spacer DNA fragments. H3K9me2 enrichment is plotted versus *S. pombe* intergenic DNA as shown in light grey (A) and DNA from the phage lambda as shown in white (B). The dark grey arrow represents the disrupted *ura4* gene. The data points on the far right represent the level of H3K9me2 enrichment observed at the pericentromeric repeats in the respective strains, as internal controls.

First, 5 kb of *S. pombe* DNA taken from a region between two divergently transcribed genes (*spcc320.02^+^* and *spcc320.03^+^*) was positioned between L5 and *ade6^+^*. This region was selected because it is one of the larger regions in the *S. pombe* genome in which known protein-coding genes are absent and because it normally lacks heterochromatic modifications [28]. Having established that this region maintains the absence of H3K9me2 when moved to the *ura4* locus in strains lacking L5 (Figure S2A), H3K9me2 levels were queried over this DNA in the presence of the L5 element. Heterochromatin was robustly enriched over the 5 kb insert DNA (Figure 2A). Strikingly, the magnitude of H3K9me2 enrichment is comparable to the level observed at *S. pombe* centromeres (Figure 2A), suggesting that H3K9me2 can reach and sustain high levels of occupancy over the entire region. This is in contrast to the pattern of heterochromatin spreading over the gene-rich *ura4* and *spbc2f12.03* neighborhoods (Figure 1A and 1C). The difference between the magnitudes of heterochromatin enrichment between these DNA sequences supports the role of *cis*-acting DNA sequences, potentially the genes themselves, in shaping the characteristics of heterochromatin domains.

Consistent with this hypothesis, there is a significant reduction in H3K9me2 enrichment coincident with the start of the *ade6^+^* gene, and the level of enrichment at this location is similar to the level observed when *ade6^+^* is adjacent to L5 (compare Figure 1A and 2A). One interpretation of these data is that only low levels of heterochromatin can exist in transcriptionally active regions. Thus, when heterochromatin spreads from the spacer DNA into the *ade6^+^* gene, the *ade6^+^* gene behaves as a heterochromatin attenuator. Alternatively, this may indicate that the extent of spreading is constrained such that heterochromatin cannot spread, with high levels of enrichment, farther than 5.6 kb from L5.

To address this latter possibility, a longer spacer sequence was selected and inserted between L5 and *ade6^+^*. Sequences from lambda phage were chosen, as they have been used in previous epigenetic studies as spacer DNA [34]. No significant enrichment was observed over the length of the 7 kb insert in the absence of L5, suggesting that these sequences do not nucleate heterochromatin on their own (Figure S2B). In contrast, when the L5 element is present, robust enrichment in H3K9me2 was observed over the length of the lambda fragment, at levels similar to that of the centromeres and the 5 kb *S. pombe* spacer fragment (Figure 2B). Moreover, when we extended our analysis to include the levels of H3K9me2 enrichment at endogenous sequences in *ura4*::L5-7kb-*ade6^+^* strains we found that it was remarkably similar to the levels observed in strains lacking spacer DNA (Figure S3). Thus, the addition of spacer DNA (up to 7 kb) does not constrain the extent of a *de novo* heterochromatin domain. Instead, our data are consistent with a model in which both the extent and magnitude of a heterochromatin domain are dictated by features of the underlying DNA sequence.

### The boundaries of *de novo* heterochromatin domains are marked by the presence of highly transcribed genes

Because endogenous sequences can influence the extent of a heterochromatin domain, we next sought to determine the factors that mediate the interaction of DNA sequence and chromatin. Based on the observation that some barriers require formation of a transcription complex [22],[27],[29],[35],[36], we investigated the relationship between domain size and transcriptional activity.

The level of transcriptional activity within both the *ura4* and the *spbc2f12.03* regions could be assessed using previously reported data sets [29],[37]. Transcriptional activity was inferred from both the steady state levels of mRNA and the level of RNA Polymerase II (Pol II) and RNA Polymerase III (Pol III) enrichment at the promoter. Between the two regions, there were five loci that were transcriptionally exceptional: one gene that was transcribed by Pol III and four genes with unusually high levels of Pol II transcriptional activity (Table 2) [29],[37].

**Table 2:** Genes within the *de novo* heterochromatin domains are highly expressed and enriched in Pol II.

Gene	Steady state mRNA levels^1^	RNA Pol II Enrichment^1^
A	1769	1.14
B	761	0.91
C	10588	3.41
E	618	0.76
F	377	
G	296	0.64
H	942	0.88
1	8746	3.28
2	347	0.64
3	1885	0.88
	1845	1.47
4	9130	3.81
5	2220	0.88
6	9810	4.75
7	364	0.80
ade6+	5472	0.91

1 From [37].

Transcription levels at the wild type *ura4^+^* and *spbc2f12.03^+^* loci.

The *ura4* genomic neighborhood includes a Pol III-transcribed tDNA^Gly^ (gene D in Figure 1A), which is coincident with an H3K9me2 gap. This gap could be attributed to general nucleosome depletion or, alternatively, to nucleosomes shielded from H3K9me2 modification by the Pol III transcription complex. Supporting the former hypothesis, tDNA genes are generally depleted of nucleosomes when compared to the genome average [38]. To distinguish between these two possibilities, an antibody to the C-terminus of histone H3 was used to characterize nucleosome occupancy surrounding to the tDNA^Gly^ gene. Indeed, the level of H3 enrichment at the tDNA^Gly^ was reduced relative to sequences in the surrounding neighborhood (Figure S1B), indicating that the observed H3K9me2 gap is due to decreased nucleosome occupancy surrounding the tDNA^Gly^.

The *ura4* genomic neighborhood includes the gene *spcc330.06^+^*(gene C in Figure 1A), which is highly expressed and enriched in Pol II (at the 94^th^ percentile genome-wide) at its promoter (Table 2) [37]. This gene is located within a striking transition in H3K9me2 enrichment from 14-fold to <2-fold enrichment over a distance of only 2.7 kb (Figure 1A). In contrast to the nucleosome gap discussed above, this transition marks a boundary of heterochromatin enrichment and cannot be explained by reduced nucleosome occupancy (Figure S1B). We hypothesize that this gene may behave as a heterochromatin barrier and, more broadly, that highly expressed genes in general may be effective heterochromatin barriers.

Within the *spbc2f12.03* genomic neighborhood, three genes are highly transcribed (genes 1,4 and 6 in Figure 1C). One of these genes (gene 6) is distal to the boundary of the *de novo* heterochromatin domain, and as such is uninformative. However, genes 1 and 4 (Figure 1B) are located at boundaries of the *de novo* heterochromatin domain, consistent with the hypothesis that highly expressed genes weaken and/or stop the spread of *de novo* heterochromatin.

### Introduction of a gene within spacer DNA attenuates heterochromatin spreading independent of level of transcription

**Figure 3:**
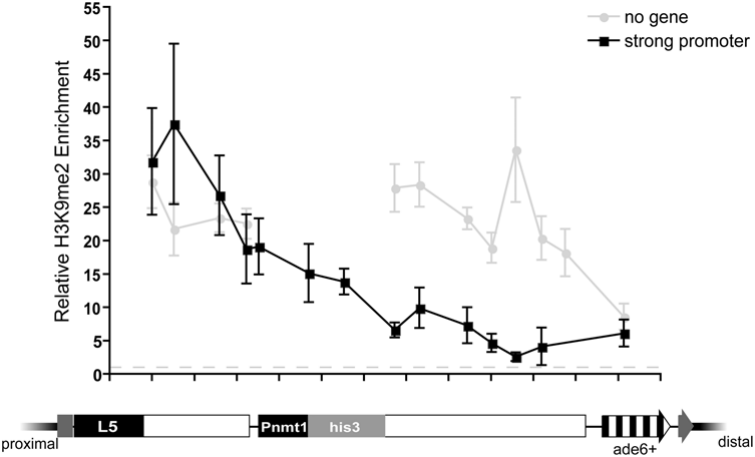
The presence of Pnmt1-his3 attenuates heterochromatin spreading. H3K9me2 enrichment in is shown for strains in which Pnmt1-*his3^+^* has been inserted within lambda (black) or containing uninterrupted spacer DNA (grey).

To directly test whether the presence of transcribed genes can influence the extent of a *de novo* heterochromatin domain, we constructed a chimeric reporter gene composed of the strong, repressible, *nmt1^+^* promoter driving expression of the *his3^+^* open reading frame (Pnmt1-*his3^+^*) [39]. This construct was then inserted within the 7 kb spacer fragment, and heterochromatin spreading was monitored over the spacer sequences and the inserted gene. As expected, H3K9me2 was highly enriched over the spacer DNA proximal to Pnmt1-*his3^+^*, consistent with the levels observed in uninterrupted spacer strains (Figure 3). However, the magnitude of H3K9me2 enrichment decreases over the Pnmt1-*his3^+^*sequences and remains reduced over the distal portion of the spacer DNA (Figure 3). These data demonstrate that the insertion of genic sequences within the spacer DNA attenuates the spread of heterochromatin and further support the hypothesis that the presence of genes within the *ura4* and *spbc2f12.03* neighborhoods limits heterochromatin spreading. It is interesting, however, that the Pnmt1-*his3^+^* construct, despite being more highly transcribed than *spcc330.06^+^* (Figure S5A), does not exhibit complete barrier activity (Figure 3), suggesting that factors other than high levels of transcriptional activity are required for complete barrier activity.

Because the presence of genes antagonizes heterochromatin spreading, we sought to determine whether a high level of transcriptional activity is required for attenuator activity. To test this we took advantage of an engineered allele of the *nmt1^+^* promoter that results in reduced transcription efficiency [40], and cultured these strains in medium containing thiamine, which results in further repression of the *nmt1^+^* promoter [39]. Despite a ∼570 fold decrease in expression the weakened Pnmt1-*his3^+^* gene still exhibited significant attenuation ability, indistinguishable from the strongest allele (Figure S5B). Thus, other features of the *nmt1^+^* promoter may serve to attenuate the spread of heterochromatin. Indeed, the region of the promoter that is required for thiamine repression binds a protein complex independent of thiamine conditions [41]. This protein complex, or other complexes that localize to the promoter independent of thiamine and transcription efficiency, may serve to attenuate the spread of heterochromatin.

### Increased L5-copy number does not alter the heterochromatin domain

**Figure 4:**
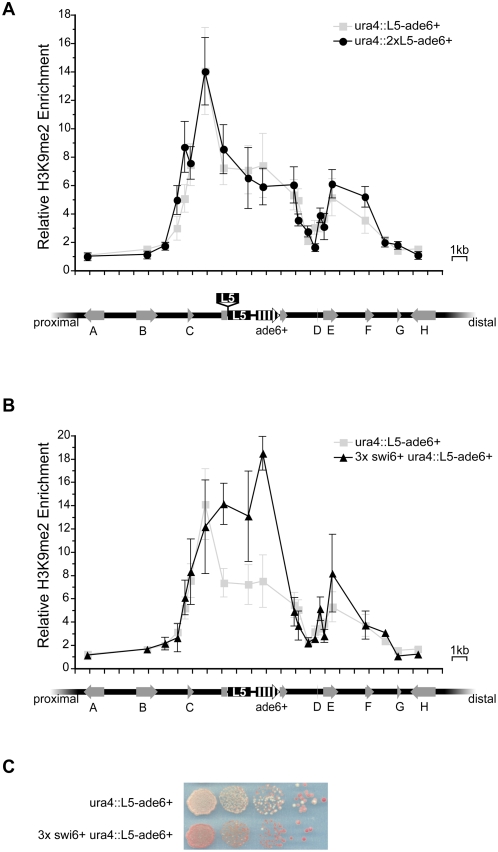
The *de novo* heterochromatin domain is shaped by the dosage of trans-acting factors. (A) H3K9me2 enrichment in the presence of an additional copy of L5 is shown in black circles. The extra copy of L5 is shown above the *ura4* genomic region, with insertion site indicated, compared to strains bearing only one copy of L5 (light grey squares). (B) H3K9me2 enrichment in strains bearing extra copies of the *swi6^+^* gene, shown in black triangles. (C) Serial dilution analysis of *ade6^+^* expression in wild type and 3× *swi6^+^* strains.

Having demonstrated the impact of genome sequence on the extent of spreading from a heterochromatin-nucleating sequence, we wanted to determine whether changes to the sequence content, in terms of L5 copy number, would alter the properties of a *de novo* heterochromatin domain. Thus, two copies of L5 were inserted in tandem at the *ura4* locus. The magnitude and extent of heterochromatin enrichment in these strains was markedly similar to strains bearing one copy of L5 (Figure 4A), suggesting that the copy number of L5 does not notably enhance heterochromatin enrichment or spreading within a *de novo* domain.

### The boundaries of the *de novo* heterochromatin domain are insensitive to increased dosage of *swi6^+^*

We also wanted to address the possible role of *trans*-acting factors in regulating the extent of the heterochromatin domain, either by competition with other heterochromatic regions for limiting heterochromatin components [42] or by competition between heterochromatic and euchromatic factors for the same nucleosome substrate [43]. We hypothesized that increasing the dosage of heterochromatin proteins (or reducing the amount of competing factors) should result in the expansion of a heterochromatin domain [9], [42]–[45].

Swi6p is a dosage-dependent modifier of heterochromatin levels at the *S. pombe* mating-type locus as well as a limiting factor in heterochromatin formation [46],[47]. Thus, we analyzed the magnitude and extent of the *ura4 de novo* heterochromatin domain in strains bearing three copies of *swi6^+^* [47]. We confirmed that the level of *swi6^+^* mRNA is increased by 2.7-fold in these strains (data not shown). While the local magnitude of H3K9me2 proximal to L5 was increased in these strains (Figure 4B), the increased dosage of *swi6^+^* did not result in the expansion of the heterochromatin domain. Consistent with the increased level of H3K9me2 enrichment, *ade6^+^* expression was further reduced in these strains, resulting in an increased proportion of red colonies (Figure 4C and Figure S4). In contrast, increased *swi6^+^* dosage did not significantly affect expression of other genes within the *ura4^+^* neighborhood (Figure S4). This suggests that, while the level of Swi6p influences both the local concentration of H3K9me2 and the level of gene expression, the extent of the *de novo* heterochromatin domain is not sensitive to increased dosage of *swi6^+^*.

### Functional distinction between local heterochromatin formation and spreading over spacer DNA

**Figure 5:**
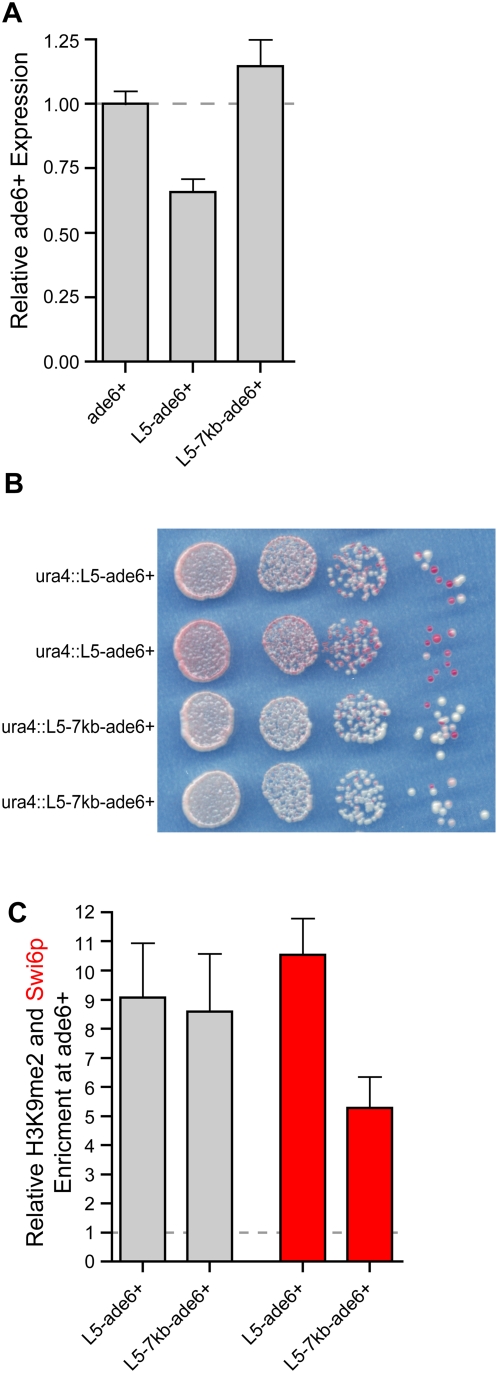
Local versus spreading over spacer DNA exerts different effects on *ade6^+^* expression. (A) Levels of *ade6^+^* expression in control (*ura4*::*ade6^+^*) strains compared to both *ura4*::L5-*ade6^+^* and *ura4*::L5-7kb-*ade6^+^*strains. (B) Serial dilution assay comparing the extent of *ade6^+^* expression in both *ura4*::L5-*ade6^+^* and *ura4*::L5-7kb-*ade6^+^*strains. (C) The level of H3K9me2 enrichment at *ade6^+^* in *ura4*::L5-*ade6^+^* and *ura4*::L5-7kb-*ade6^+^* strains is shown as gray bars. The level of Swi6p enrichment is plotted in red.

In the absence of known transcribed elements, H3K9me2 spreads unattenuated over distances at least up to 7 kb (Figure 2B), resulting in a consistent level of H3K9me2-enrichment at *ade6^+^* independent of the presence of spacer DNA (Figure 5C). What remains to be addressed is whether the level and the stability of gene silencing differ between strains containing spacer DNA versus *ura4*::L5-*ade6^+^*.

To explore this question, we compared the levels of *ade6^+^*expression by qRT–PCR and found that when *ade6^+^*was located 7 kb away from the L5 element, distal to the lambda spacer, silencing was no longer observed despite the presence of H3K9me2 (Figure 5A and 5C). This finding was confirmed using the phenotypic *ade6^+^* assay, which revealed a much lower level of silencing in *ura4*::L5-7kb-*ade6^+^* strains (Figure 5B). These data suggest that, even when the levels of H3K9me2-enrichment are similar (Figure 5C), heterochromatin formed proximal to L5 and heterochromatin formed over spacer DNA can have different effects on gene expression.

We suspected that the differences in silencing could be attributed to the levels of Swi6p at the *ade6^+^* gene in *ura4*::L5-*ade6^+^* as compared to *ura4*::L5-7kb-*ade6^+^* strains. To address this hypothesis, we assessed the level of Swi6p enrichment over the lambda spacer DNA and distal *ade6^+^* gene. We observed a significant decrease in Swi6p enrichment relative to the level of H3K9me2 across spacer and *ade6^+^* DNA when compared to *ura4*::L5-*ade6^+^* and *spbc2f12.03*::*ura4*::L5-*ade6^+^* strains, as well as to other heterochromatic loci (Figure S6 and Figure 5C). This reduction is consistent with the decreased levels of silencing and could be a function of long distance spreading or a sequence-dependent affect of spacer DNA.

**Figure 6:**
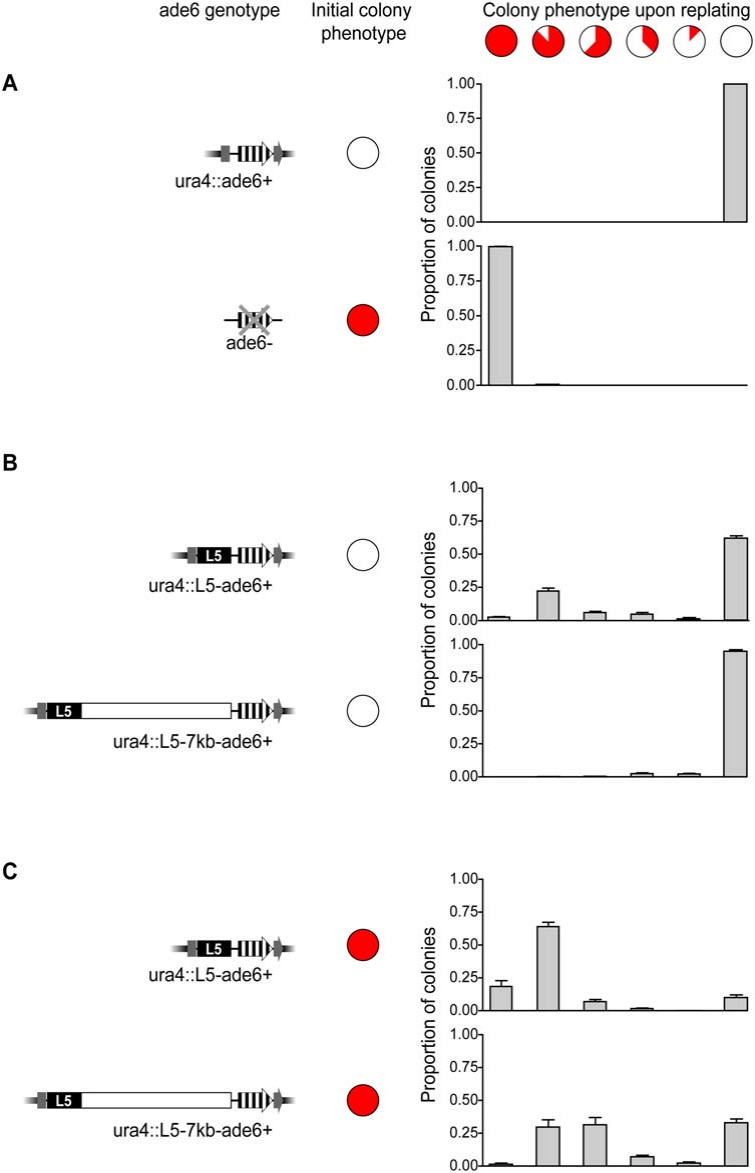
The extent and stability of *ade6^+^* silencing is altered in spacer strains. (A–C) Colonies were selected on the basis of the initial colony phenotype (either all entirely red or entirely white) for each of the given genotypes. After a 24-hour period of growth, strains were re-plated and the phenotypes of the resultant colonies were scored and classified based on the proportion of the colony that exhibited silencing, as indicated schematically above the graphs. Intermediate silencing phenotypes included sectoring and homogenous, intermediate levels of pigmentation. The graphs below represent the proportion of colonies in each class based on genotype and initial colony color.

In addition to the total level of gene silencing, another manner in which the reduced levels of Swi6p in spacer strains could affect gene expression is by altering the stability of gene repression. When transgenes are placed within the centromere, or at locations throughout the mating type locus, their phenotypic stability (silenced or expressed) can vary with location and Swi6p dosage [31],[32],[46],[47]. To address whether the silenced and expressed states are stable to equivalent degrees in cases of local (high levels of Swi6p) versus spreading over spacer DNA (reduced levels of Swi6p) we chose colonies that were either silenced or expressed, as determined by their *ade6^+^* expression phenotype (ie. entirely red or entirely white, respectively). The stability of the silenced state was determined by the proportion of progeny that exhibited silencing after a period of overnight growth. We examined the phenotypic stability of *ade6^+^* and *ade6^−^* controls, and as expected, the progeny maintained the appropriate phenotype (Figure 6A). However, when expressed colonies were chosen from *ura4*::L5-*ade6^+^* strains, only ∼62% maintained the completely expressed phenotype, while the remaining colonies switched to a partially or completely silenced phenotype. This is in stark contrast to the *ura4*::L5-7kb-*ade6^+^* strains, in which 95% of the progeny maintained the expressed state (Figure 6B), suggesting that the establishment of silencing (i.e., switching from an *ade6^+^* expressed state to a silenced state) occurs less often when *ade6^+^* is distal to 7 kb of spacer DNA and less enriched in Swi6p, despite comparable levels of the epigenetic mark H3K9me2.

When silenced colonies were selected from *ura4*::L5-*ade6^+^* strains, ∼18% of the progeny exhibited phenotypes indicative of complete silencing and 90% exhibited at least partial silencing. In contrast, silenced colonies from *ura4*::L5-7kb-*ade6^+^* strains were less likely to give rise to progeny that exhibited complete or partial silencing (∼1% and 69%, respectively), suggesting that maintenance of silencing is also less frequent when *ade6^+^* is separated from L5 by spacer DNA and reduced in Swi6p localization (Figure 6C). These data provide evidence that the level of Swi6 impacts the establishment and maintenance of silencing, despite consistent levels of H3K9me2.

## Discussion

Ectopic gene silencing and/or heterochromatin formation has previously been studied in mammalian systems [48]–[51]. Ectopic X inactivation, for example, has been shown to affect gene expression on a large scale [52]. Typically, however, the complex nature of the mammalian genome restricts the focus of these studies to local heterochromatin formation and single gene repression. In this study, the compact nature of the *S. pombe* genome and our ability to robustly query for the presence of heterochromatin allowed us to rigorously test the response of multiple DNA sequences to encroaching heterochromatin. Our data demonstrate a clear effect of genomic sequence in shaping both the extent and magnitude of a heterochromatin domain and demonstrate that, while the eukaryotic genome is permissive to the negative transcriptional effects of heterochromatin, euchromatic sequences can counteract encroaching heterochromatin.

### *de novo* heterochromatin domains are shaped by DNA sequences that vary in their ability to promote or antagonize heterochromatin spreading

**Figure 7:**
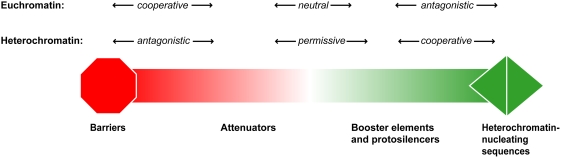
The continuum of DNA sequence, its effects on heterochromatin spreading, and the balance between opposing heterochromatic and euchromatic forces. The range of DNA sequences and the magnitude of their interaction with DNA is depicted along a gradient ranging from heterochromatin barriers (red) to heterochromatin nucleating sequences (green). The italic text above the figure describes the effect of a specific DNA sequence on both heterochromatin and euchromatin. Below the figure is a classification system that subdivides the continuum.

The relationship between the size of a heterochromatin domain and the presence of specific heterochromatin barriers has been previously established in a number of eukaryotic organisms [53]. Our study extends this conclusion, demonstrating that DNA sequences exert a range of effects on heterochromatin domains. For example, the *ade6^+^* gene dampens heterochromatin enrichment independent of both genomic location and distance from L5, but is insufficient to completely stop heterochromatin spreading (Figures 1A, 1C, 2A, and 2B). In contrast, intergenic and spacer DNA sequences promote the assembly of robust H3K9me2. We propose that there is a spectrum of effects, ranging from antagonistic to cooperative, that genomic sequence can exert on heterochromatin (Figure 7). This model incorporates the complexity and context dependence of genomic sequence and its relationship to heterochromatin and is applicable to sequences in yeast, as seen here, or in more complex genomes, as will be discussed below.

For this model, we have subdivided the discrete extremes of DNA sequences noted previously (that is, heterochromatin-nucleating sequences and heterochromatin barriers) into subclasses that include attenuators, neutral elements and protosilencers/boosters. While this is helpful for purposes of discussion, we do not wish to impose strict definitions, especially in light of data from this study, suggesting that particular sequences may be placed at multiple points along the continuum, depending on their context.

### Heterochromatin antagonists: barriers and attenuators

Our data confirm that heterochromatin can spread from L5 in both directions over euchromatic DNA, resulting in a *de novo* heterochromatin domain encompassing multiple endogenous genes and altering gene expression (Figure 1A and 1C, Table 1). However, gene repression within the *de novo* domain is moderate, at most about 50%. The incomplete silencing observed within *de novo* heterochromatin domains, as well as the boundaries of these domains, may be a consequence of the factors present within euchromatic domains that antagonize the propagation of heterochromatin.

The boundaries of *de novo* heterochromatin domains are marked by three highly transcribed genes, implicating Pol II transcription in barrier activity (Figure 1A and 1C, Table 2) [37]. The *ade6^+^* gene is also transcribed and enriched in Pol II, albeit to a lesser extent than the three putative barriers. These four sequences may rely on transcription to counteract the spread of heterochromatin from the L5 heterochromatin-nucleating sequences. However, high levels of transcription are insufficient for complete barrier activity (Figure 3). Furthermore, we find that, in the case of Pnmt1-*his3^+^*, the presence of genes can attenuate the spread of heterochromatin independent of the level of transcription (Figure S5B). We conclude that DNA sequences modify heterochromatin spreading through the sequence-dependent recruitment of other mediating factors, such as transcription complexes, and dictate whether a sequence behaves as a true barrier or falls in the range of heterochromatin attenuators (Figure 7). These findings are consistent with previous results implicating transcription factors and promoters with barrier activity [35], [54]–[58]. Sequence could also influence heterochromatin directly, as is the case with some examples of nucleosome positioning [59] or could reflect selective pressure to maintain domain boundaries (Table 1).

In addition to protein-coding genes, the *ura4 de novo* heterochromatin domain includes a tDNA^Gly^ gene (discussed below) and non-coding RNAs (Figure 1A). The *ura4* locus is not unique in its transcriptional makeup, as recent studies have provided insight into the vast amount of transcription occurring in the *S. pombe* genome outside of canonical protein coding genes [60],[61]. Additionally, the *ura4* neighborhood also includes solo long terminal repeats (LTRs) [62]. How these features interact with heterochromatin spreading, and whether they shape the formation of *de novo* heterochromatin domains warrants further genome-wide studies.

Transcription by Pol III complexes has an established relationship with barrier activity in yeast genomes [27],[29],[35],[36]. A tDNA^Ala^ within the *S. pombe* centromere 1 prevents the spread of heterochromatin into the abutting domain of centromeric chromatin. In contrast, the tDNA^Gly^ gene is not coincident with the domain boundary of the ectopic heterochromatin domain formed at *ura4^+^*; however it is deficient in H3K9me2 enrichment, due to the absence of a nucleosome(s) (Figure 1A and Figure S1B). Nucleosome depletion has been shown previously to restrict heterochromatin spreading [63]; in this context, the nucleosome gap may weaken the spread of heterochromatin, resulting in the gradual attenuation observed distal to the tDNA^Gly^. We suggest that, like the *ade6^+^* gene, the tDNA^Gly^ behaves as heterochromatin attenuator in our experimental system. It is interesting to note that, while other tDNAs substituted at the centromere recapitulate barrier activity, re-positioning of tDNA^Ala^ at a euchromatic locus resulted in an attenuation of heterochromatin spreading, but not complete barrier activity [27]. Together, these data establish a mechanistic link between heterochromatin barriers and attenuators, and implicate genomic context as an additional factor in determining where a sequence falls along the continuum of effects on heterochromatin (Figure 7).

### DNA sequences prevent expansion of heterochromatin domains

Whether the effect of DNA sequence could be abrogated by increased dosage of heterochromatin proteins was also examined. Increased *swi6^+^* resulted in increased levels of H3K9me2 over sequences adjacent to L5, as well as enhanced repression of *ade6^+^*, consistent with an increase in local heterochromatin (Figure 4B). However, this change in heterochromatin enrichment is not accompanied by an expansion of the domain. Further expansion of the domain is likely prevented by the barrier and attenuator activity of adjacent sequences, indicating that these sequences are robust to the increasing magnitude of heterochromatin in these strains. This is the also the case with models of PEV in mouse where enhancing heterochromatin formation is insufficient to cause PEV when a transgene is flanked by chromatin insulators [64]. Alternatively, enhanced propagation of heterochromatin could be limited by selection against increased silencing of genes within the *de novo* heterochroamtin domain.

### Active and passive heterochromatin advocates: protosilencers, boosters, and neutral sequences

As heterochromatin antagonists are characterized by different strengths, we propose that DNA sequences also differ in their ability to initiate or promote heterochromatin spreading. The identification of protosilencers, sequences that can actively contribute to gene silencing but only in specific “silencing-conducive” environments [65], supports this hypothesis. DNA sequences that are permissive to heterochromatin spreading can be conceptually subdivided into those that rely on active mechanisms, like those above, and those that passively allow heterochromatin but do not actively propagate the heterochromatic state (Figure 7). The spacer and *S. pombe* intergenic fragments may fall into this class of sequence elements. Both sequences allow formation of large heterochromatin domains with levels of H3K9me2 enrichment similar to that observed at the centromere (Figure 2A and 2B). Alternatively, these sequences may contain elements that enhance heterochromatin spreading, and thus would belong in the former class of sequences that actively promote heterochromatin spreading.

Interestingly, while high levels of H3K9me2 are sustained over the length of the lambda spacer DNA, the ratio of Swi6p/H3K9me2 is reduced, relative to both genome-wide data and data from the *ura4* and *spbc2f12.03 de novo* heterochromatin domains (Figure 5C and Figure S6) [28]. The reduced levels of Swi6p correlate with reduced ability to establish and maintain silencing at *ade6^+^* when compared to *ura4*::L5-*ade6^+^* strains (Figure 6). These data suggest that lambda spacer DNA exerts a sequence-specific effect on the associated heterochromatin domain that results in reduced levels of gene repression.

### Genome sequence affects chromatin state in higher eukaryotes, as well as fission yeast

The spreading of heterochromatin from L5 shares at least conceptual similarities with the spreading of gene silencing and, presumably, facultative heterochromatin from an ectopic X inactivation center in mammalian systems [52],[66]. Furthemore, as we demonstrate in fission yeast, genome sequence is also implicated in the organization of chromatin on the mammalian X chromosome [67]. The inactive X chromosome is organized in alternating domains of genes that are subject to inactivation (silenced) and domains of genes that escape from X inactivation (expressed) [68],[69], as well as by domains of different types of heterochromatin [70],[71]. A CTCF site on the mouse inactive X chromosome, located within such a transition region, exhibits insulator activity in transgene assays [23], thus implicating DNA sequence in maintaining the boundaries of expression domains. Moreover, the presence of specific DNA features on the X chromosome can be used to accurately predict whether a gene will be subject to, or escape from, X inactivation [72],[73]. However, as with the intergenic and spacer fragments in this study, it is unknown whether the sequences correlated with gene silencing passively permit the silent state, or whether they actively promote the propagation of gene silencing. Finally, LINE-1 elements have been proposed to behave as protosilencers, or booster elements, relaying transcriptional inactivation from sites of nucleation [72],[74]. While such evidence points to the importance of DNA sequence in regulating domains of gene expression on the X chromosome, the presence of barriers and other sequences in mammals has yet to be addressed fully.

## Materials and Methods

### Fission yeast strains

The genotypes for strains used in this study are as listed (Table S1). Fission yeast media were prepared using standard procedures [75]. For repression of the *nmt1* promoter 15 uM thiaimine was added [76]. A strain bearing the *ade6^DN/N^* allele (a loss of function mutation created by a 153 bp deletion of the *ade6^+^* open reading frame [77]) was generated (Kfy539) and was transformed via electroporation (1.5 kV, 200Ω, 25uF) on a BioRad Gene Pulser II. Transformed cells were selected on PMG media lacking adenine [75]. Colonies derived from strain Kfy539 were then patched onto media containing 2 g/L of 5-fluoro-orotic acid (FOA) (MP Biomedicals) to select for disruption of *ura4^+^*. The resulting strains were screened, using Southern analysis, for appropriate integration of *ade6^+^*. Additionally, BW17 transformants were screened by Southern blot for the maintenance of the 7 kb lambda DNA fragment. At least three independent transformants of each genotype were maintained (with the exception of the random integrant, Kfy812) and used for further analysis. All transformants were then crossed into a *swi6^+^* strain and the *ura4::*L5-*ade6^+^* allele was selected for on the basis of FOA resistance. To create *swi6+333* strains, *ura4::*L5-*ade6^+^* strains were crossed into SPG1232 (Shiv Grewal) [47]. To create *ura4*::L5-7kb::(Pnmt1-*his3^+^*)-*ade6^+^* the Pnmt1-*his3^+^* construct was transformed into Kfy589, colonies were selected for on the basis of growth on media lacking histidine. After integration within lambda was confirmed by Southern analysis, these strains were crossed into a *swi6^+^* strain.

### Plasmids

To create plasmid BW5, *ade6* ^+^ was amplified from *S. pombe* genomic DNA using primers BWP34F and BWP34Rb (Table S2) to add StuI, SpeI, ClaI, and BglII sites to the 5′ end of the product and Sac1, Sma1 and Stu1 sites to the 3′ end. The PCR product was then digested with StuI and inserted into the StuI site of *ura4^+^* in pUC13/18. The *ade6^+^* open reading frame and upstream region were sequenced to ensure no mutations had been introduced during cloning.

Plasmid BW7 was constructed through digestion of YL317 with SpeI and ClaI and subsequent purification of the L5-containing fragment [27]. L5 was then inserted into the SpeI/ClaI site of BW5. Plasmids BW32 and BW34 contain 4.9 kb of *S. pombe* intergenic DNA taken from between SPCC320.02 and SPCC320.03 inserted into the BglII site of BW5 and BW7, respectively. The intergenic fragment was digested from the cosmid SPCC320 using XbaI, subcloned into pUC13/18, and then digested with BamHI before inserting into the appropriate plasmid. Plasmids BW30 and BW17 were created by digesting the lambda phage genome (NEB) with BamHI and purifying the 7.2 kb fragment, which was then ligated into the BglII sites of BW5 and BW7, respectively. To create BW20 an additional copy of L5, as a BamHI–BglII fragment, was inserted into the BglII site of BW7.

Plasmids BWP40 and BWP41 were created by replacing the GFP ORF with *his3^+^* within the plasmids pFA6a-kanMX6-P3nmt1-GFP and pFA6a-kanMX6-P41nmt1-GFP, respectively (A gift from Jian-Qiu Wu) [78]. A subfragment of the lambda spacer DNA was liberated from BW17 by digestion with BglII and cloned into pUC1318. The Pnmt1-*his3^+^* containing fragment was then inserted within the PstI site in the lambda fragment.

### Confirming and mapping random integrants

To identify random integrants that did not disrupt the *ura4* locus, we selected transformants on the basis of growth on PMG–adenine and death on FOA. These strains were then confirmed via Southern blot to have a single *ade6^+^* insertion and the site of integration was mapped using an inverse PCR protocol modified from [79]. Genomic DNA (2 µL) was digested with MboI or Nde1 and incubated for 3.5 hours at 37°C. The digest was heat inactivated at 65°C for 20 minutes. 2 µL of the digest was added to a standard ligation reaction (T4 ligase, NEB) and incubated overnight at room temperature. Inverse PCR was performed using primers E367/BWP89F for the Nde1 digest and BWP37F/BWP32F for the Mbo1 digest. The PCR products were purified and sequenced using the PCR primers listed above.

### Serial dilution analysis and scoring of *ade6^+^* phenotypes

Strains were grown overnight with shaking in YES media at 32°C and diluted to a concentration of 1e6 cells/mL. Cultures were diluted serially (1∶9) and plated on YES media lacking adenine.

To assess the stability of silencing, colonies that were scored as either completely white or completely red were identified using a Leica MZ7.5 microscope and grown for 24 hours in YES media before plating on YE plates lacking adenine.

For both protocols, plates were grown for three nights at 32°C and shifted to 4°C for 24 hours before photographing or counting.

### Real time RT–PCR

Total nucleic acid was isolated from logarithmically growing cells in YES media at 32°C, and was then subjected to DNAse treatment and RT–PCR using oligodT as a primer. Expression was analyzed by quantitative PCR using SYBR Green on a Bio-rad myCycler, using primers specific to the wild type copy of *ade6^+^* (BWP85F/R). Levels of mRNA from *ade6^+^*, and other genes queried, were expressed relative to *act1^+^*(BWP74F/R). The standard curve was generated using genomic DNA isolated from strain Kfy1. In order to be included in this study a PCR experiment had to have a PCR efficiency between 90–110% and a correlation coefficient >0.99.

### Chromatin immunoprecipitation

The H3K9me2 ChIP protocol was adapted from [80]. Logarithmically growing cells from control and experimental strains were treated with 1% paraformaldehyde for 15 minutes. The cell wall was then destroyed through bead beating twice for two minutes in buffer containing protease inhibitors. The resulting material was then sheared to an average fragment size of 600 bp using sonication. Chromatin preps were then subdivided into three tubes: an input sample that was used to check shearing, an IP sample to which protein beads and antibodies to H3K9me2 (from Takeshi Urano) were added, and a mock sample to which only protein beads were added. The mock and IP samples were incubated overnight, and the beads were isolated and subjected to a series of washes. Finally, DNA was purified from all three samples (IP, mock, and input) with phenol-chloroform extraction and ethanol precipitation using glycogen as a carrier.

H3 ChIPs were preformed as above using an antibody to H3 (abcam 1791).

Swi6p ChIPs were performed using the above protocol modified from [81]. 2.5e8 cells were shifted to room temperature for two hours prior to fixation. Cells were fixed with 3% paraformaldehyde for 30 minutes at room temperature. 1 µL of antibody (from Shiv Grewal) was incubated with the IP sample overnight, prior to incubation with protein beads for two hours.

Quantitative PCR was used to assay levels of query/*act1^+^* in IP reactions relative to a no-antibody control.

## Supporting Information

Figure S1 The *ura4* genomic region is not enriched in H3K9me2 in the absence of L5, and exhibits varible nucleosome occupancy. (A) The *ura4* locus is depicted, and assayed for the presence of H3K9me2 (black) and Swi6p (grey), in the absence of the L5 element. (B) Nucleosome occupancy was characterized using an antibody to the c-terminus of histone H3. The data are expressed relative to the nucleosome occupancy at the *act1+* locus.
(3.00 MB TIF)

Figure S2 *S. pombe* intergenic and lambda spacer fragments do not recruit H3K9me2 in the absence of L5. H3K9me2 enrichment over *S. pombe* intergenic (A) and lambda (B) sequences in the absence of L5. The intergenic spacer DNA is in duplicate copies in the genome (at the *ura4* locus as well as its endogenous locus) thus no enrichment is represented by a value of 2.
(1.88 MB TIF)

Figure S3 Heterochromatin spreading in the *ura4* locus is unaffected by the presence of lambda spacer DNA. Analysis of H3K9me2 in the *ura4* locus in *ura4::L5-7kb-ade6^+^* strains (black) and *ura4::L5-ade6^+^* strains. To facilitate comparison between the two strains H3K9me2 enrichment for the *ura4::L5-ade6^+^* is depicted with a gap over the lambda insert.
(1.93 MB TIF)

Figure S4 Increased *swi6^+^* copy number results in decreased *ade6^+^* expression but does not alter expression of other genes within the *de novo* heterochromatin domain. Steady state mRNA levels are depicted relative to *ura4::ade6^+^* strains for wild type strains (grey) and strains bearing 3 copies of *swi6^+^* (black).
(1.59 MB TIF)

Figure S5 Insertion of a gene within lambda attenuates heterochromatin independent of the level of transcription. (A) Levels of steady state mRNA relative to *act1^+^*. *his3^+^* mRNA was isolated from highly transcribed (strong *nmt1* allele, no thiamine) and weakly transcribed conditions (weak *nmt1* allele, thiamine) in *swi6- ura4::L5-7kb::*(Pnmt1-*his3^+^*)-*ade6^+^* strains. For comparison the level of *ade6^+^* and *spcc330.03^+^* mRNA are shown for *ura4::ade6^+^* strains. (B) Relative H3K9me2 enrichment for strongly and weakly transcribed conditions.
(2.72 MB TIF)

Figure S6 The ratio of Swi6p/H3K9me2 is reduced over spacer DNA. Scatter plot of the levels of Swi6p/H3K9me2 for heterochromatic regions genomewide [28], or within *de novo* heterochromatin domains.
(1.47 MB TIF)

Table S1 Strains used in this study.
(0.04 MB PDF)

Table S2 Primers used in this study.
(0.08 MB PDF)
