# Supplementary figures and images for: The Impact of Local Genome Sequence on Defining Heterochromatin Domains
Source: PLoS Genet. 2009 Apr 10;5(4):e1000453. doi: 10.1371/journal.pgen.1000453 (PMC2659443; doi:10.1371/journal.pgen.1000453)

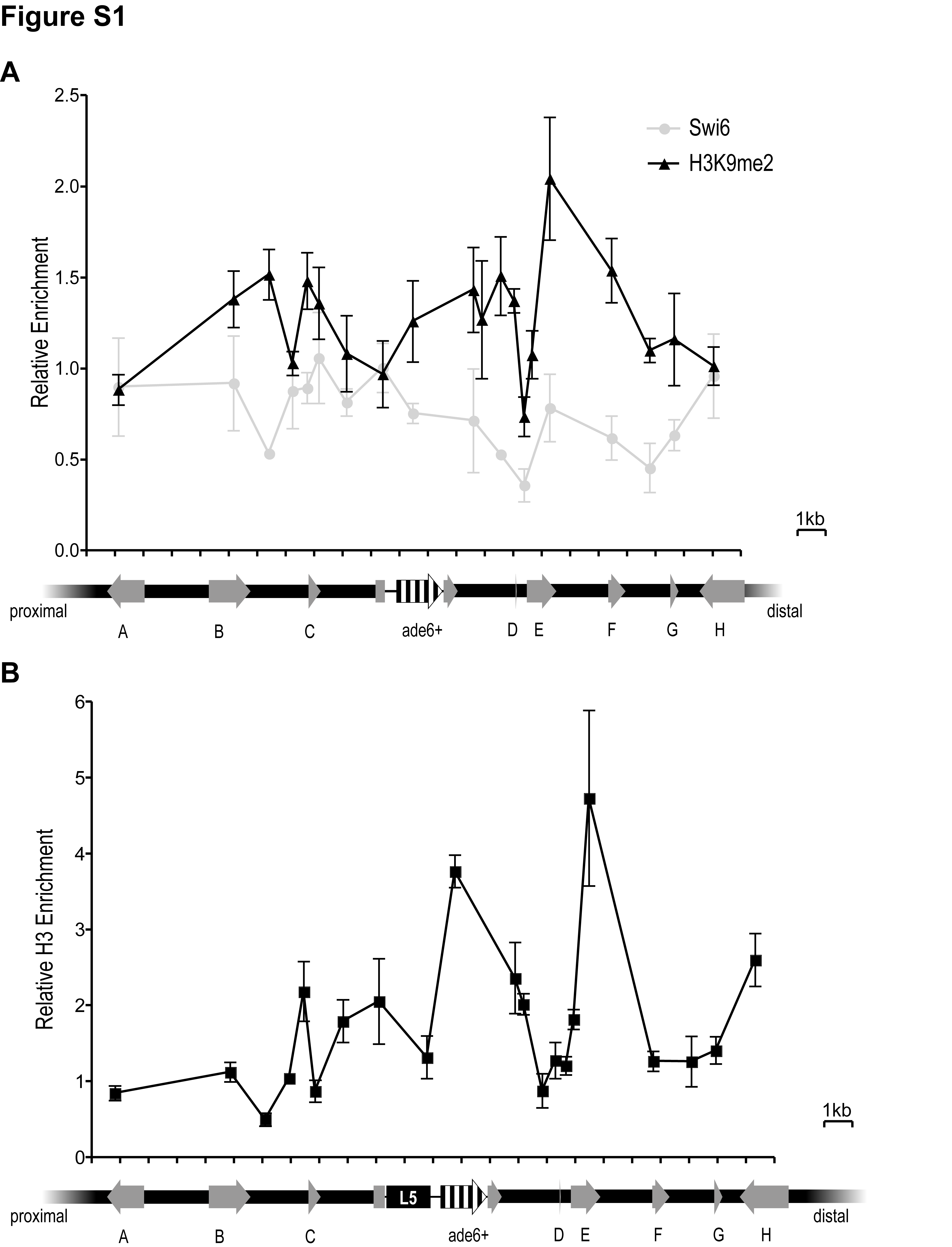

Supplement: Figure S1 — The ura4 genomic region is not enriched in H3K9me2 in the absence of L5, and exhibits varible nucleosome occupancy. (A) The ura4 locus is depicted, and assayed for the presence of H3K9me2 (black) and Swi6p (grey), in the absence of the L5 element. (B) Nucleosome occupancy was characterized using an antibody to the c-terminus of histone H3. The data are expressed relative to the nucleosome occupancy at the act1+ locus. (3.00 MB TIF) [file pgen.1000453.s001.tif]

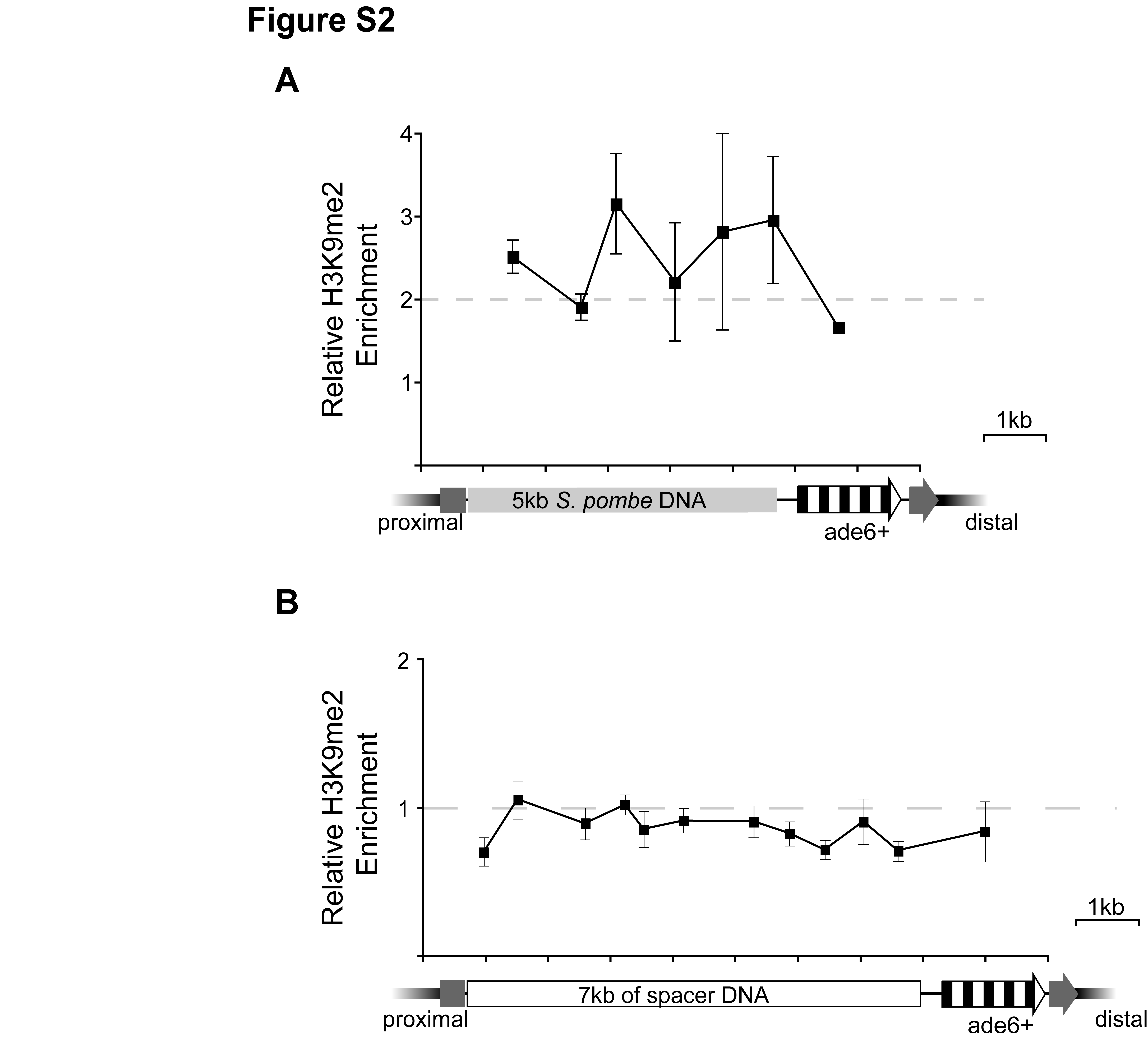

Supplement: Figure S2 — S. pombe intergenic and lambda spacer fragments do not recruit H3K9me2 in the absence of L5. H3K9me2 enrichment over S. pombe intergenic (A) and lambda (B) sequences in the absence of L5. The intergenic spacer DNA is in duplicate copies in the genome (at the ura4 locus as well as its endogenous locus) thus no enrichment is represented by a value of 2. (1.88 MB TIF) [file pgen.1000453.s002.tif]

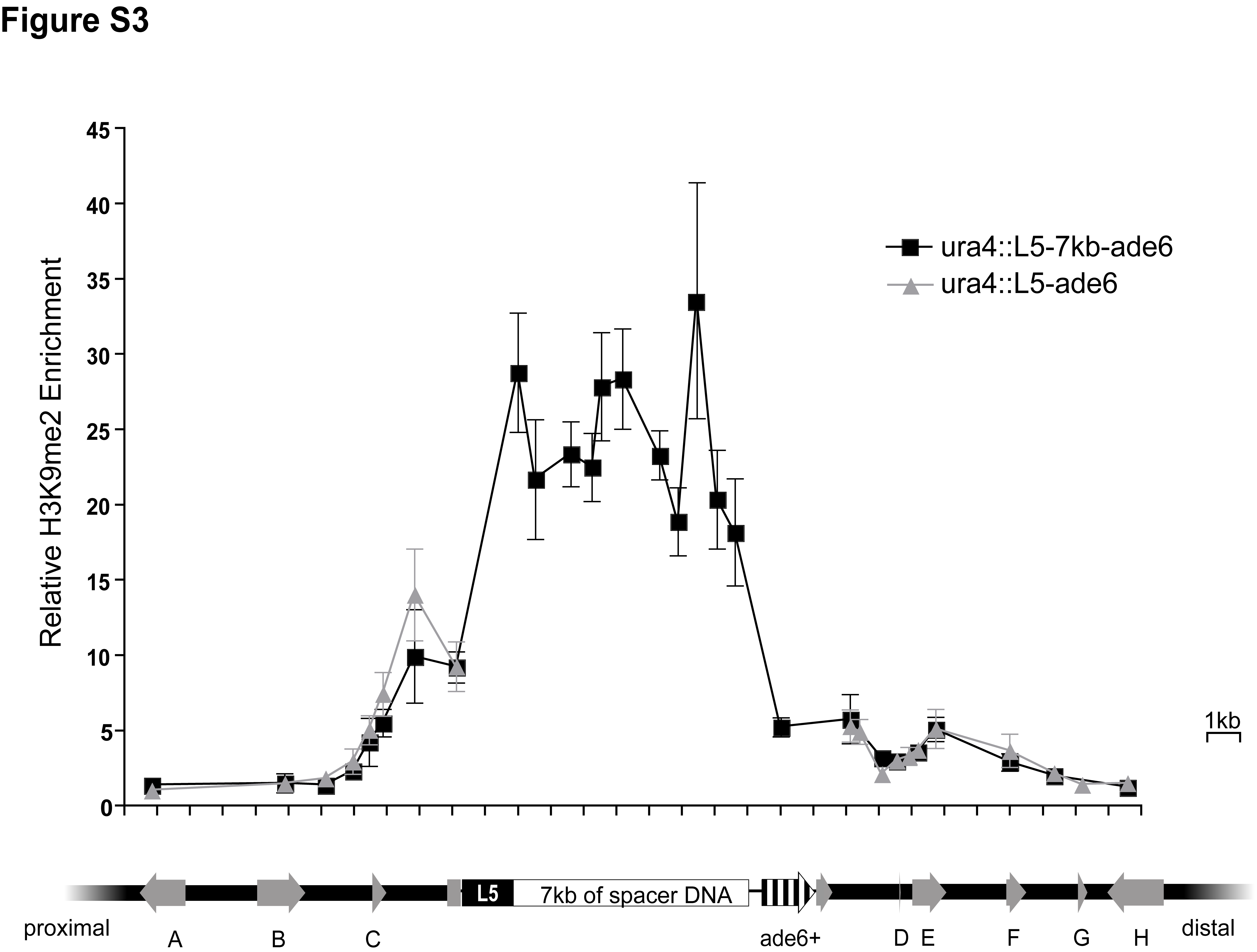

Supplement: Figure S3 — Heterochromatin spreading in the ura4 locus is unaffected by the presence of lambda spacer DNA. Analysis of H3K9me2 in the ura4 locus in ura4::L5-7kb-ade6+ strains (black) and ura4::L5-ade6+ strains. To facilitate comparison between the two strains H3K9me2 enrichment for the ura4::L5-ade6+ is depicted with a gap over the lambda insert. (1.93 MB TIF) [file pgen.1000453.s003.tif]

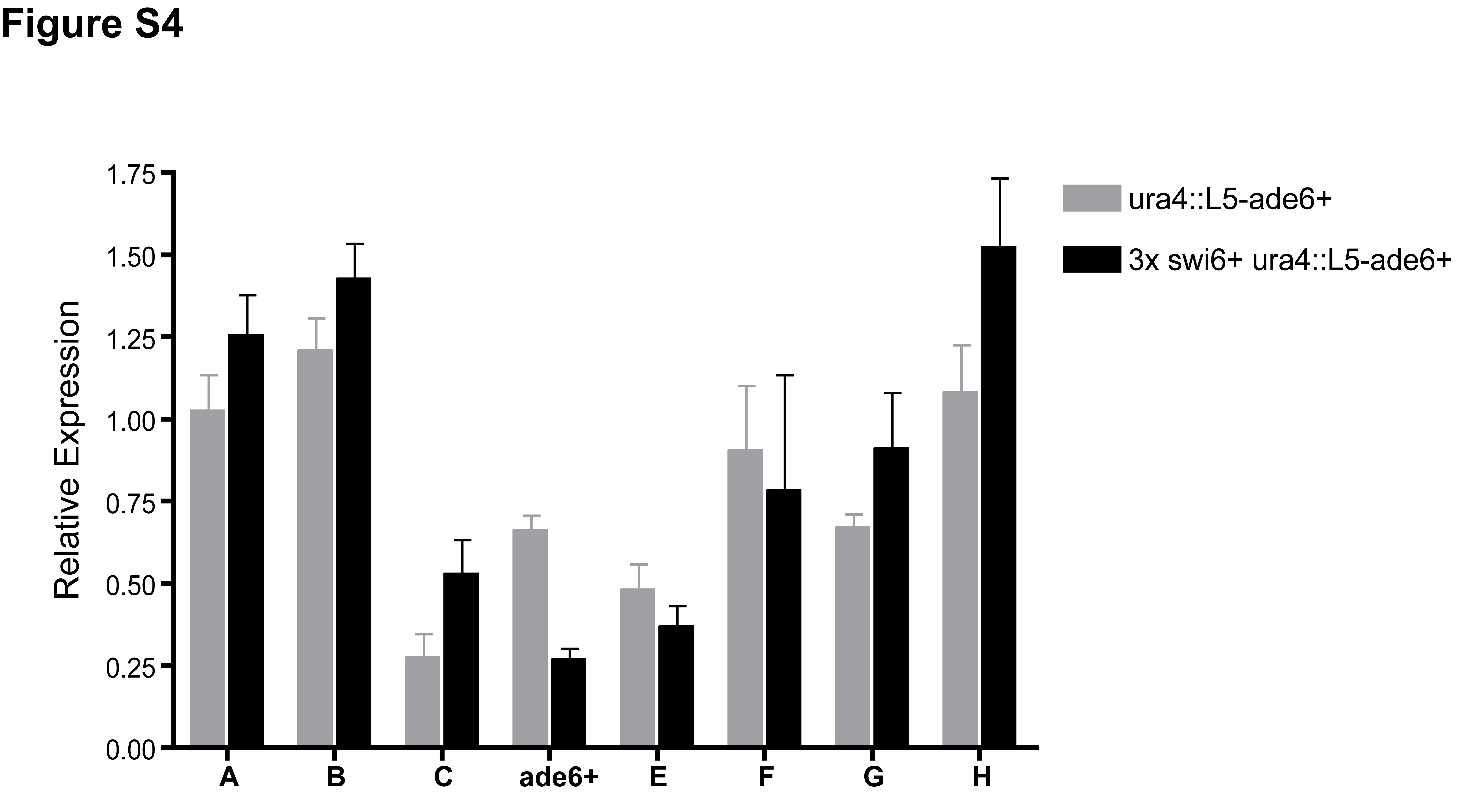

Supplement: Figure S4 — Increased swi6+ copy number results in decreased ade6+ expression but does not alter expression of other genes within the de novo heterochromatin domain. Steady state mRNA levels are depicted relative to ura4::ade6+ strains for wild type strains (grey) and strains bearing 3 copies of swi6+ (black). (1.59 MB TIF) [file pgen.1000453.s004.tif]

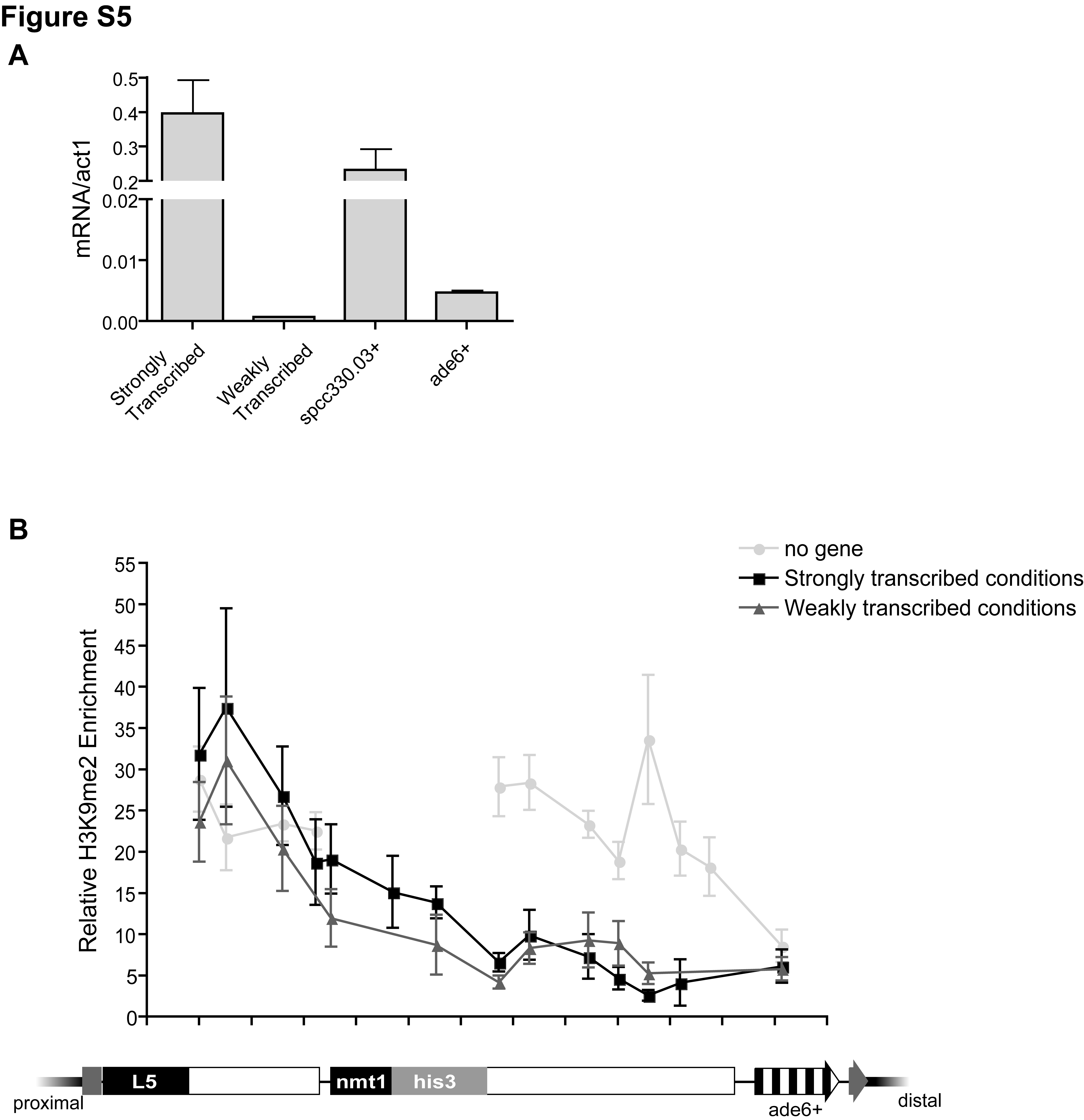

Supplement: Figure S5 — Insertion of a gene within lambda attenuates heterochromatin independent of the level of transcription. (A) Levels of steady state mRNA relative to act1+. his3+ mRNA was isolated from highly transcribed (strong nmt1 allele, no thiamine) and weakly transcribed conditions (weak nmt1 allele, thiamine) in swi6- ura4::L5-7kb::(Pnmt1-his3+)-ade6+ strains. For comparison the level of ade6+ and spcc330.03+ mRNA are shown for ura4::ade6+ strains. (B) Relative H3K9me2 enrichment for strongly and weakly transcribed conditions. (2.72 MB TIF) [file pgen.1000453.s005.tif]

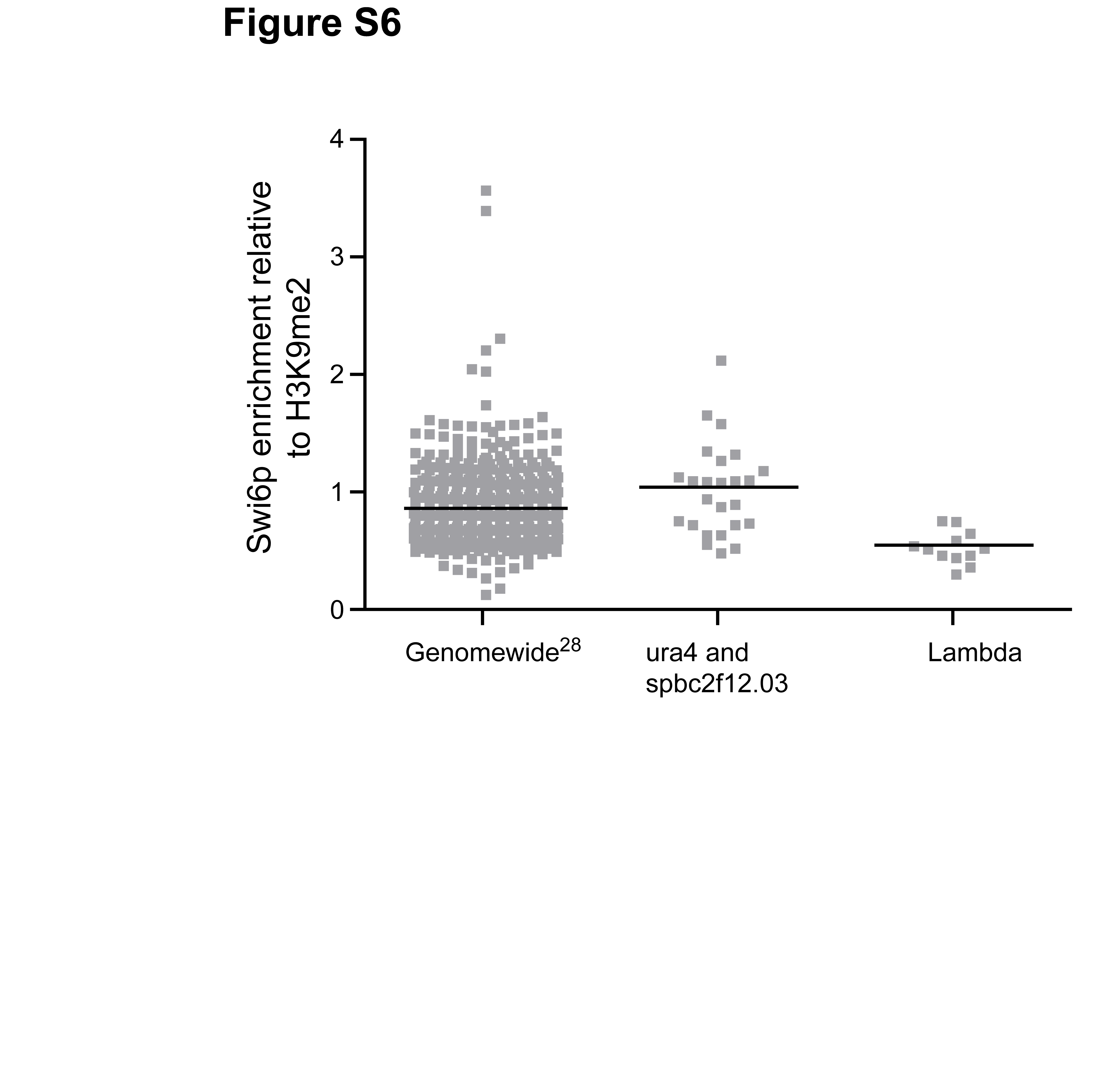

Supplement: Figure S6 — The ratio of Swi6p/H3K9me2 is reduced over spacer DNA. Scatter plot of the levels of Swi6p/H3K9me2 for heterochromatic regions genomewide [28], or within de novo heterochromatin domains. (1.47 MB TIF) [file pgen.1000453.s006.tif]
