# Supplementary material for: The Impact of Local Genome Sequence on Defining Heterochromatin Domains
Source: PLoS Genet. 2009 Apr 10;5(4):e1000453. doi: 10.1371/journal.pgen.1000453 (PMC2659443; doi:10.1371/journal.pgen.1000453)
Supplement: Table S1 — Strains used in this study. (0.04 MB PDF) [file pgen.1000453.s007.pdf]

Table S1

Strain	Genotype	Source
Kfy1	h- ura4D18	R. Allshire (FY106)
Kfy2	h- ade6-210	R. Allshire (FY92)
Kfy3	h+ ura4D18 ade6-210 leu1-32 arg3D his3D	R. Allshire (FY1645)
Kfy4	h- ura4D18 ade6-210 leu1-32 arg3D his3D	R. Allshire (FY1646)
Kfy450	h+ ade6DN/N leu1-32 ura4D18 his3D arg3D	R. Allshire (FY3749)
Kfy687	swi6+333 (leu2+) KΔ::ura4+ leu1-32 ura4DS/E his2 ade6-210	S. Grewal (SPG1232)
Kfy501	h+ ade6DN/N his3D leu1-32 arg3D	This Study
Kfy616	h+ ura4::ade6 ade6DN/N his3D leu1-32 arg3D	This Study
Kfy617	h+ ura4::ade6 ade6DN/N his3D leu1-32 arg3D	This Study
Kfy618	h+ ura4::ade6 ade6DN/N his3D leu1-32 arg3D	This Study
Kfy619	h+ ura4::ade6 ade6DN/N his3D leu1-32 arg3D	This Study
Kfy620	h+ ura4::L5-ade6 ade6DN/N his3D leu1-32 arg3D	This Study
Kfy621	h+ ura4::L5-ade6 ade6DN/N his3D leu1-32 arg3D	This Study
Kfy622	h+ ura4::L5-ade6 ade6DN/N his3D leu1-32 arg3D	This Study
Kfy623	h+ ura4::L5-ade6 ade6DN/N his3D leu1-32 arg3D	This Study
Kfy812	spbc2f12.03::ura4::L5-ade6 ade6DN/N his3D leu1-32	This Study
Kfy847	swi6+333 (leu2) ura4::L5-ade6 ade6DN/N his3D leu1-32 arg3D	This Study
Kfy901	swi6+333 (leu2) ura4::L5-ade6 ade6DN/N his3D leu1-32 arg3D	This Study
Kfy902	swi6+333 (leu2) ura4::L5-ade6 ade6DN/N his3D leu1-32 arg3D	This Study
Kfy937	ura4::5kbPombe-ade6 ade6DN/N his3D leu1-32 arg3D	This Study
Kfy1011	ura4::5kbPombe-ade6 ade6DN/N his3D leu1-32 arg3D	This Study
Kfy1044	ura4::5kbPombe-ade6 ade6DN/N his3D leu1-32 arg3D	This Study
Kfy1012	ura4::L5-5kbPombe-ade6 ade6DN/N his3D leu1-32 arg3D	This Study
Kfy1013	ura4::L5-5kbPombe-ade6 ade6DN/N his3D leu1-32 arg3D	This Study
Kfy1014	ura4::L5-5kbPombe-ade6 ade6DN/N his3D leu1-32 arg3D	This Study
Kfy935	ura4::7kb-ade6 ade6DN/N his3D leu1-32 arg3D	This Study
Kfy991	ura4::7kb-ade6 ade6DN/N his3D leu1-32 arg3D	This Study
Kfy992	ura4::7kb-ade6 ade6DN/N his3D leu1-32 arg3D	This Study
Kfy641	ura4::L5-7kb-ade6 ade6DN/N his3D leu1-32 arg3D	This Study
Kfy644	ura4::L5-7kb-ade6 ade6DN/N his3D leu1-32 arg3D	This Study
Kfy647	ura4::L5-7kb-ade6 ade6DN/N his3D leu1-32 arg3D	This Study
Kfy724	h+ ura4::2xL5-ade6 ade6DN/N his3D leu1-32 arg3D	This Study
Kfy725	h+ ura4::2xL5-ade6 ade6DN/N his3D leu1-32 arg3D	This Study
Kfy726	h+ ura4::2xL5-ade6 ade6DN/N his3D leu1-32 arg3D	This Study
Kfy1130	ura4::L5-7kb::(P3nmt1-his3)-ade6 ade6DN/N his3D leu1-32	This Study
Kfy1132	ura4::L5-7kb::(P3nmt1-his3)-ade6 ade6DN/N his3D leu1-32	This Study
Kfy1133	ura4::L5-7kb::(P3nmt1-his3)-ade6 ade6DN/N his3D leu1-32	This Study
Kfy1137	ura4::L5-7kb::(P41nmt1-his3)-ade6 ade6DN/N his3D leu1-32	This Study
Kfy1140	ura4::L5-7kb::(P41nmt1-his3)-ade6 ade6DN/N his3D leu1-32	This Study
Kfy1141	ura4::L5-7kb::(P41nmt1-his3)-ade6 ade6DN/N his3D leu1-32	This Study
