# Supplementary material for: The Impact of Local Genome Sequence on Defining Heterochromatin Domains
Source: PLoS Genet. 2009 Apr 10;5(4):e1000453. doi: 10.1371/journal.pgen.1000453 (PMC2659443; doi:10.1371/journal.pgen.1000453)
Supplement: Table S2 — Primers used in this study. (0.08 MB PDF) [file pgen.1000453.s008.pdf]

Table S2

Oligo	Sequence	Purpose
E367	CCATAAAGATGCAAAGTTGCACC	iPCR
BWP32F	TTCCTACTGCCATCAAAGCA	iPCR
BWP34F	gatatcaggcctactagtgcatgcatcgatagatct TCAACTTGGTGGTGAGGTAAACG	Construction of BW5
BWP34Rb	aggcctcccgggagctcCAGCACATTATTCGGGGGG	Construction of BW5
BWP37F	TGATATGAGCCCAAGAAGCA	iPCR
BWP38F	CCCTACATGGAATGGAATCG	*his3*$^+$ qRT-PCR
BWP38R	GCAAACAGCAAGGTTTGGAT	*his3*$^+$ qRT-PCR
BWP41F	TTTACCAAGGCCTTTGATGC	*pmp20*$^+$ qRT-PCR
BWP41R	CCTTGTCAGCGGAGGAAATA	*pmp20*$^+$ qRT-PCR
BWP45F	TTCAACGAGACATGCGAAAC	*mug135*$^+$ qRT-PCR
BWP45R	TCGAGGCAAATTAGGGTCAG	*mug135*$^+$ qRT-PCR
BWP50F	TGGCCTGAATATGTTAATGAGAGA	7.2kb lambda ChIP
BWP50R	GCAATAGCTGATAATCGATGCAC	7.2kb lambda ChIP
BWP51F	TACGTTGCAGGTTGCTTTCA	7.2kb lambda ChIP
BWP51R	TTAACCGCCCTATTCTCTCG	7.2kb lambda ChIP
BWP53F	TTACTATGTAAACACCAGGCATGA	7.2kb lambda ChIP
BWP53R	GAATGAAAAGTGATATACTGGAATGTT	7.2kb lambda ChIP
BWP54F	GTCATCTGCGAGGCTGTTCT	7.2kb lambda ChIP
BWP54R	TCGATGGAAAAACTTTTCTCTTTAC	7.2kb lambda ChIP
BWP55F	CCGACCAGAACACCTTGC	7.2kb lambda ChIP
BWP55R	CCCACAGTACCCAATGATCC	7.2kb lambda ChIP
BWP56F	ATAAAGCACCAACGCCTGAC	7.2kb lambda ChIP
BWP56R	AACACAAGAGCAGCTTGAGGA	7.2kb lambda ChIP
BWP57F	GCGAGATTACAAAGTTACCTGTCA	7.2kb lambda ChIP
BWP57R	TGCACCATTCTGAGATGTTTTT	7.2kb lambda ChIP
BWP58F	GCGATAAGTGGACCCAACTC	7.2kb lambda ChIP
BWP58R	CATCTGCTCACGGTCAAAGT	7.2kb lambda ChIP
BWP59F	GCGCACTACTGGCTGGTTAC	7.2kb lambda ChIP
BWP59R	GCCCATGACAGGAAGTTGTT	7.2kb lambda ChIP
BWP60F	TTCGATCTCCAGCACATCAG	7.2kb lambda ChIP
BWP60F	AGGCCCATAGCTTCCTGTTT	7.2kb lambda ChIP
BWP61F	CGGTTTGTCAGGGAAGTTGT	7.2kb lambda ChIP
BWP61R	ATGGCTTCAGAACAGGCATT	7.2kb lambda ChIP
BWP71F	TCAGGAAGCGAATTCTAAGGA	*spcc330.03*$^+$ qRT-PCR
BWP71R	AAAGCATCCCAATCTTGTTGA	*spcc330.03*$^+$ qRT-PCR
BWP74F	ACTCTGTCTGGATTGGTGGA	*act1*$^+$ qRT-PCR
BWP74R	AACGATACCAGGTCCGCTCT	*act1*$^+$ qRT-PCR
BWP77F	GGCTGACAAAAGCGAAAAAG	*330.07*$^+$ qRT-PCR
BWP77R	AATGCCACTTCCGCTAAAAA	*330.07*$^+$ qRT-PCR
BWP84F	CGGCATCGCTTGTACTTTTT	centromere
BWP84R	GACGGAACCAAATGATGTGA	centromere
BWP85F	CATGGAAATTGCAGTGATGG	*ade6*$^+$ qRT-PCR
BWP85R	CGAGCAGGGGCATATACTAAA	*ade6*$^+$ qRT-PCR
BWP86F	TTGATGCCAGACCGTAATGA	*ura4* locus ChIP
BWP86R	TCTGACATGGCATTCCTCAA	*ura4* locus ChIP
BWP89F	CTGCGGTAGCGATATCATCA	iPCR
BWP91F	TAAGGGGAAGGGAAAGGAAA	5kb IG ChIP

BWP91R	GCTAGCCGTTTCTCATCTCG	5kb IG ChIP
BWP92F	TTGAACGATGGAAAACAGTGA	5kb IG ChIP
BWP92R	TTTCAGACTCCCTACCCTTTTT	5kb IG ChIP
BWP93F	CAAACGATAGCAACTATGTCAGC	5kb IG ChIP
BWP93R	GCCTGCGAAAACTTCAATCT	5kb IG ChIP
BWP94F	AAAAACTGCAAGCACGAGGT	5kb IG ChIP
BWP94R	AAACGGCCGACAGATTAAAA	5kb IG ChIP
BWP95F	TTGCATCTAATGAATGTTTTTGC	5kb IG ChIP
BWP95R	TAGGGGGATAAGTGGAACGA	5kb IG ChIP
BWP96F	GGATAGGAATTTCCCGGTGT	5kb IG ChIP
BWP96R	TGCATAGGCCGATGAAAAGT	5kb IG ChIP
BWP98F	TGCATCATGAGAAAGGGAGA	*ura4* locus ChIP
BWP98R	GATTTGGGAAAACGGATGTG	*ura4* locus ChIP
BWP99F	TTTTGAATACGCCGTTACTCC	*ura4* locus ChIP
BWP99R	GATCCGACAGCAACCATTTT	*ura4* locus ChIP
BWP100F	GGCCTTAGGTAAAAAGCATCG	*ura4* locus ChIP
BWP100R	TGAGCCCAAGAAGCAATTTT	*ura4* locus ChIP
BWP102F	CACAATATCGGTGCAAATAGG	*ura4* locus ChIP
BWP102R	TCAGGAGGAATTATTTTGGGAAT	*ura4* locus ChIP
BWP103F	AAGATGCATTTCCATTATAATCCTC	*ura4* locus ChIP
BWP103R	TCATTTTTGCTTATCGATCACTG	*ura4* locus ChIP
BWP104F	CGTATATGGGGTTGGCTTGT	*ura4* locus ChIP
BWP104R	CGACAAGGTACAGTTTAGCAATG	*ura4* locus ChIP
BWP107F	TTTATTCAACACAGCCAAGGAA	*ura4* locus ChIP
BWP107R	CATACAAATGAATATTCGACAATGTG	*ura4* locus ChIP
BWP108F	TGACCGTAGTCGTAAACTCGAA	*spcc330.19*$^+$ qRT-PCR
BWP108R	AAAGCAAGACAAGCGGTATGA	*spcc330.19*$^+$ qRT-PCR
BWP111F	GCCTGGTGATTCAATCTTCAAA	*rhp7*$^+$ qRT-PCR
BWP111R	TTGCCCAAATTTGATTAGCC	*rhp7*$^+$ qRT-PCR
BWP114F	AAACAACCTTGAAAATGAATCAAA	*ura4* locus ChIP
BWP114R	AACGAAGTGATGATGTTTTTCTTG	*ura4* locus ChIP
BWP118F	ACGCTGAGAGGGAGTTTGAA	*ura4* locus ChIP
BWP118R	CCAACATGCCTGTAAGTAGTTTT	*ura4* locus ChIP
BWP120F	CCCGTATTTCTCAAAATAAATCTCA	*ura4* locus ChIP
BWP120R	CACCAAAGCCGATTTATTCC	*ura4* locus ChIP
BWP121F	GCTAGAAAGGAAAGCATTGGAA	*ura4* locus ChIP
BWP121R	TCAAAAGCCCTTTTCTTGGA	*ura4* locus ChIP
BWP128F	AGCATCTACAACAAATTTGACTTTT	*alg11*$^+$ qRT-PCR
BWP128R	GCGTTCGAGGTGAAGAAGAC	*alg11*$^+$ qRT-PCR
BWP135F	AACCAACGACATCATGGGTAG	L5-*ade6*$^+$ junction
BWP135R	CAGGCTGGATTTCGTTTACC	L5-*ade6*$^+$ junction
BWP159F	GTATTCTGGGCCTCCATGTC	lambda-Pnmt1 junction
BWP159R	TGAAACAACCGCTGATGACT	lambda-Pnmt1 junction
BWP160F	TTTCAATCTCATTCTCACTTTCTGA	Pnmt1-*his3*$^+$ junction
BWP160R	GCACAGCGATAAGGCTGAAG	Pnmt1-*his3*$^+$ junction
BWP165F	TTACGCACGTTTCATTTCCA	*spbc2f12.03*$^+$ locus ChIP
BWP165R	GGCTCATCTAGGTCAAGTTCG	*spbc2f12.03*$^+$ locus ChIP
BWP166F	GATGCCTATTATCCGTGAAGC	*ceg1*$^+$ qRT-PCR
BWP166R	TTGATTGCAGGAGCATCATC	*ceg1*$^+$ qRT-PCR
BWP167F	AACCATCAACACGTTGGTCA	*rpl802*$^+$ qRT-PCR
BWP167R	AAAGACCGGTTCTACGAGCA	*rpl802*$^+$ qRT-PCR
BWP168F	CAAATATGCGGGCTCTCAA	*spbc2f12.03*$^+$ locus ChIP
BWP168R	TTCGTGGCTCATTCTCATTTT	*spbc2f12.03*$^+$ locus ChIP
BWP169F	TGCCCGTGAATTTAAGAAGG	*spbc2f12.03*$^+$ locus ChIP

BWP169R	TTCATTCAAATCAATAGCATCATT	*spbc2f12.03*$^+$ locus ChIP
BWP170F	AACCATGCAAACAAAGCAGA	*spbc2f12.03*$^+$ locus ChIP
BWP170R	GAAGGTTGTGGCTTATGTTCAG	*spbc2f12.03*$^+$ locus ChIP
BWP171F	CGATCGTCCGAAAATCAGTC	*spbc2f12.05*$^+$ qRT-PCR
BWP171R	TTCATTGAAGCTTACTGGCAAA	*spbc2f12.05*$^+$ qRT-PCR
BWP172F	TTTGCAAAAAGGCAAGGAAT	*spbc2f12.03*$^+$ locus ChIP
BWP172R	TAAATAAACTTTACTCAGTGTTGTTGG	*spbc2f12.03*$^+$ locus ChIP
BWP173F	CTGAATAGCGGACCATTGCT	*spbc2f12.03*$^+$ locus ChIP
BWP173R	AAGTGTCCAAACCAGTCACAA	*spbc2f12.03*$^+$ locus ChIP
BWP174F	CAAGGCTTGCTGCTCTTTTT	*spbc2f12.03*$^+$ locus ChIP
BWP174R	GGATGATGGAGAGCCTGAAA	*spbc2f12.03*$^+$ locus ChIP
BWP175F	TGCCAATGCTTCTACCTTTG	*spbc2f12.03*$^+$ locus ChIP
BWP175R	TCTGTGAAAGTCCAGTTCGAATA	*spbc2f12.03*$^+$ locus ChIP
BWP176F	TTGTTTACATAGGACTGCGTTGA	*spbc2f12.03*$^+$ locus ChIP
BWP176R	AATCAATTGGCAAGCTGAGG	*spbc2f12.03*$^+$ locus ChIP
BWP177F	CGGATTTCCTTACTTGGCACT	*spbc2f12.03*$^+$ locus ChIP
BWP177R	GGATCTTAGGTACTAGGGCTGGA	*spbc2f12.03*$^+$ locus ChIP
BWP178F	CATGCCTTTATTTCCCCAAA	*mrpl7*$^+$ qRT-PCR
BWP178F	CCAAACGGGTATCTTTGGAA	*mrpl7*$^+$ qRT-PCR
BWP179F	AAATGATGGGGATGAACCAG	*byr2*$^+$ qRT-PCR
BWP179R	CCAACGGAAATGAAAAATCG	*byr2*$^+$ qRT-PCR
BWP180F	TCCATAAATGCAGGAAAGTGG	*spbc2f12.03*$^+$ locus ChIP
BWP180F	CGAGACTAAGGGAGGCAGGT	*spbc2f12.03*$^+$ locus ChIP
BWP181F	TGGTTCAGCTCACTTCTCCA	*spbc2f12.03*$^+$ locus ChIP
BWP181R	TTGGCAATATAATGCTGTTTCG	*spbc2f12.03*$^+$ locus ChIP
